# Genes related to emphysema are enriched for ubiquitination pathways

**DOI:** 10.1186/1471-2466-14-187

**Published:** 2014-11-29

**Authors:** Sergey Stepaniants, I-Ming Wang, Yves Boie, James Mortimer, Brian Kennedy, Mark Elliott, Shizu Hayashi, Honglin Luo, Jerry Wong, Leanna Loy, Silvija Coulter, Christopher J Roberts, James C Hogg, Don D Sin, Gary O’Neill, Michael Crackower, Melody Morris, Peter D Paré, Ma’en Obeidat

**Affiliations:** Covance Genomics Laboratory, Indianapolis, USA; Merck Research Laboratory, Rahway, USA; University of British Columbia Centre for Heart and Lung Innovation, St Paul’s Hospital, 1081 Burrard St, Vancouver, V6Z 1Y6 BC Canada

**Keywords:** Pulmonary emphysema, Surface area to lung volume ratio, Gene expression, Transcriptional analysis, mRNA, Cigarette smoking, Protein ubquitination

## Abstract

**Background:**

Increased small airway resistance and decreased lung elasticity contribute to the airflow limitation in chronic obstructive pulmonary disease (COPD). The lesion that corresponds to loss of lung elasticity is emphysema; the small airway obstruction is due to inflammatory narrowing and obliteration. Despite their convergence in altered physiology, different mechanisms contribute to these processes. The relationships between gene expression and these specific phenotypes may be more revealing than comparison with lung function.

**Methods:**

We measured the ratio of alveolar surface area to lung volume (SA/V) in lung tissue from 43 smokers. Two samples from 21 subjects, in which SA/V differed by >49 cm^2^/mL were profiled to select genes whose expression correlated with SA/V. Significant genes were tested for replication in the 22 remaining subjects.

**Results:**

The level of expression of 181 transcripts was related to SA/V ( p < 0.05). When these genes were tested in the 22 remaining subjects as a replication, thirty of the 181 genes remained significantly associated with SA/V (P < 0.05) and the direction of association was the same in 164/181. Pathway and network analysis revealed enrichment of genes involved in protein ubiquitination, and western blotting showed altered expression of genes involved in protein ubiquitination in obstructed individuals.

**Conclusion:**

This study implicates modified protein ubiquitination and degradation as a potentially important pathway in the pathogenesis of emphysema.

**Electronic supplementary material:**

The online version of this article (doi:10.1186/1471-2466-14-187) contains supplementary material, which is available to authorized users.

## Background

The pathology of chronic obstructive pulmonary disease (COPD) includes 2 main components; chronic obstructive bronchiolitis with fibrosis which causes narrowing and ultimately obliteration of small airways, and emphysema with enlargement of airspaces and destruction of lung parenchyma which causes loss of lung elasticity [1]. Most patients with COPD have both lesions although one may predominate in individual patients. Although the processes differ substantially they both lead to a common phenotype, reduced maximal expiratory flow.

One method to discover the molecular mechanisms leading to disease is to compare gene expression pattern in the organs of affected and non-affected individuals. However since the gene expression patterns that contribute to airway remodeling and to emphysema may be different, correlation of gene expression with the individual lesions, rather than with lung function, may be more revealing. The degree of emphysema can be measured microscopically as the lung surface area to volume ratio (SA/V) [2]. In the current study we identified 181 genes whose expression pattern correlated with the severity of emphysema using two samples of lung tissue with different SA/V from 21 smokers with variable degrees of airflow obstruction whose lungs were resected for small peripheral lung lesions (P < 0.05). To validate associations we examined the relationship between expression of these genes and SA/V in a separate set of 22 lung samples. 30 of the 181 genes were significantly associated with SA/V in the replication set. Gene set annotation and pathway analyses showed that genes involved in protein ubiquitination and the proteasome pathway were differentially expressed in emphysematous lung tissue. Protein analysis confirmed the abnormal accumulation of protein-ubiquitin conjugates and the altered expression of genes involved in protein ubiquitination in the lungs of obstructed smokers.

## Methods

### Subject selection and experimental design

Preoperatively, patients provided informed written consent that a portion of their lung tissue be used for research. The experimental protocols were approved by the ethics review board of the University of British Columbia and St Paul’s Hospital. Ethics approval numbers are H00-50110 and H09-00801. The strategy for selecting patients for gene profiling has been described in a previous publication [3] and is detailed in the online supplement. The frozen lung tissue samples from 43 subjects with a range of lung function were profiled. In 21 of the subjects 2 samples, which showed widely different values for SA/V, were profiled to derive relationships between gene expression and SA/V. For the remaining 22 subjects, one sample of lung tissue was profiled and served as a validation data set for the relationships between gene expression and SA/V.

### Lung function measurement

Details of lung function were described previously [3] and are contained in the online supplement. Briefly, measures of lung volumes, spirometry and the single breath diffusing capacity were measured as previously described and according to ATS standards [4]. Table 1 shows the number of patients in each GOLD category and their mean smoking history and lung function.

**Table 1:** **Subject characteristics**

GOLD stage	N	Age (yrs)	Male/Female	Pack years	FEV1 % predicted	FEV1/FVC %	DLCO % predicted	Diagnoses
Non-smokers	4	59 ± 8	2/2	O	90 ± 5	82 ± 3	71 ± 4	4 Carcinoid
Control Smokers	18	67 ± 8	11/7	38 ± 19	98 ± 13	76 ± 4	80 ± 15	6 Squamous
								7 Adeno
								4 Large cell
								1 Small cell
1	9	61 ± 11	6/3	50 ± 14	89 ± 6	66 ± 4	78 ± 21	4 Squamous
								3 Adeno
								1 Large cell
								1 Small cell
2	9	63 ± 9	6/3	50 ± 21	66 ± 7	58 ± 4	68 ± 17	1 Squamous
								5 Adeno
								1 Large cell
								1 Poorly differentiated
								1 Amyloid
3	3	67 ± 5	2/1	49 ± 12	46 ± 1	53 ± 9	56 ± 14	1 Adeno (BAC)
								1 Squamous
								1 Carcinoid

Means ± SD. An ANOVA showed no significant difference in age by GOLD category but significant differences for pack years, FEV1 % predicted, FEV1/FVC % and DLCO % Predicted. (p < 0.0001 for all comparisons) Among smokers there was no significant difference in pack years by GOLD category. Control smokers – 14/21 Current smokers, GOLD 1 - 6/9 Current smokers. GOLD 2 – 9/10 Current smokers and GOLD 3 – 2 Current smokers and one status unknown. Current smoker = within 6 months of surgical resection.

### Tissue processing

Details of tissue processing and determination of SA/V were described previously [3] and are contained in the online supplement. Briefly, immediately following resection the lung or lobe was frozen in liquid nitrogen fumes. Frozen cores of tissue (1.5 × 2 cm) were obtained from slices of frozen lung using a power driven hole saw. Frozen sections were obtained from the surface of the ½ core immediately adjacent to the portion to be used for RNA extraction. The 10 micron sections were stained with hematoxylin and eosin and the severity of emphysema in each core was measured on digital images of the sample using an in-house point counting program.

### Microarray study design and methodology

RNA from 8 GOLD 0 subjects (non-obstructed smokers) was pooled to form the reference RNA as described [3] and as detailed in the online supplement. mRNA from the specimens was profiled using Agilent’s Functional IDv2.0 array (Agilent, Santa Clara,CA). Microarray profiling was done for 23,757 probe-sets as previously described [3]. The gene expression data was deposited in Gene Expression Omnibus (GEO; http://www.ncbi.nlm.nih.gov/projects/geo/), under the accession number GSE63073. Since a “batch effect” confounded the results, we performed a very conservative phase/batch adjustment step before commencing with statistical analysis (see online supplement).

### Statistical analysis

In order to identify genes correlated to SA/V, Matlab Statistical Toolbox Analysis of Covariance (ANCOVA) model (7.0.1.24704 [R14] Service Pack 1) with parallel lines was used to fit each transcript to the SA/V ratio and simultaneously account for patient effect.


Where y_ij_ is gene expression with i =1, …21 representing the patient; and j =1, 2 the two samples of lung from each patient respectively. β_0_ stands for the overall intercept of the model, β_i_ is the intercept for each patient, α represent the common slope for the parallel lines for all patients, and ϵ_ij_ the model residual respectively. In this model the significance of the patient effect is accounted for by the variation between individual intercepts β_i_ and its corresponding patient p-value (P_patient_), while slope α and its corresponding p-value (P_slope_) represent the regression coefficient and its significance with respect to SA/V, respectively. This model searches for the correlative pattern between gene expression and SA/V common to all patients as slope α is the same for all patients. It does not model individual patient SA/V association, which would be given by the corresponding interaction effect.

Genes that have significant patient effect, i.e., vary more between than within patients, can be selected by requiring P_patient_ <0.01. Conversely, those that do not vary significantly between the patients can be picked by setting P_patient_ >0.1, for example. Genes that significantly associate with SA/V can be picked by establishing a p value cut off for the slope α. It is these latter genes which are of interest since they relate to the degree of emphysema.

This model was fit individually to all 23,757 probe-sets on the microarray. Multiplicity adjustments using Benjamini-Hochberg false discovery rate (FDR) were used to adjust raw p-values for the slope α. Although 181 genes showed nominal association with SA/V (P < 0.05), none passed FDR <0.1. However, real associations could be artificially diluted by the large number of probe sets tested in the course of the statistical analysis. One way to overcome this problem is to perform a conservative selection of candidate markers and then assess their performance in an independent replication set. This latter step is also required even for those markers which pass the FDR cutoff, and constitutes the ultimate check for the validity of the findings.

Thus, in order to identify markers for subsequent replication a combination of statistical cutoffs was used. First, more highly expressed genes that satisfied an average logIntensity > -1 cutoff were selected. Secondly, the genes were selected to be significantly associated with SA/V by requiring P_slope_ <0.05. Finally, the set was refined by filtering out those with significant patient effect, by requiring P_patient_ >0.1. The reason that the final cutoff was applied is to allow a replication of the SA/V related genes in the independent set of 22 patient samples. For these patients only a single lung sample was available and adjusting for the patient effect is not possible. Minimizing the patient effect allows a simple linear regression model to approximate the slope of the fit between each of the 181 genes and the corresponding SA/V ratios in the replication set.

### Networks and pathways enrichment analyses

To determine whether the 181 genes which were related to lung SA/V were enriched for specific biological processes or were suggestive of specific disease states we undertook pathway and network analysis using MetaCore® from GeneGo Inc. Gene IDs of the 181 transcripts were used as input for MetaCore®.

### Ubuquitination pathway analysis

In this study, we found that a number of deregulated genes are related to the process of ubiquitination or de-ubiquitination. To test if there was concordant dysregulation of this pathway at the protein level, we performed Western analyses on extracts from an additional group of lung samples. Based on commercial availability of the antibodies (see below), we selected 9 genes that were differentially regulated as a function of SA/V from the list. The genes that were included in this analysis are shown in Additional file 1: Table S6 with a brief description of their potential role. For the protein quantification a separate subset of 20 individual lung samples were examined (See online supplement for details). In addition to the measurement of protein levels, the proteosome activity of the lung tissue from 5 of the cases and 5 controls was measured.

Details of the Western blotting and proteosome activity assays are available in the online supplement.

## Results

**Figure 1:**
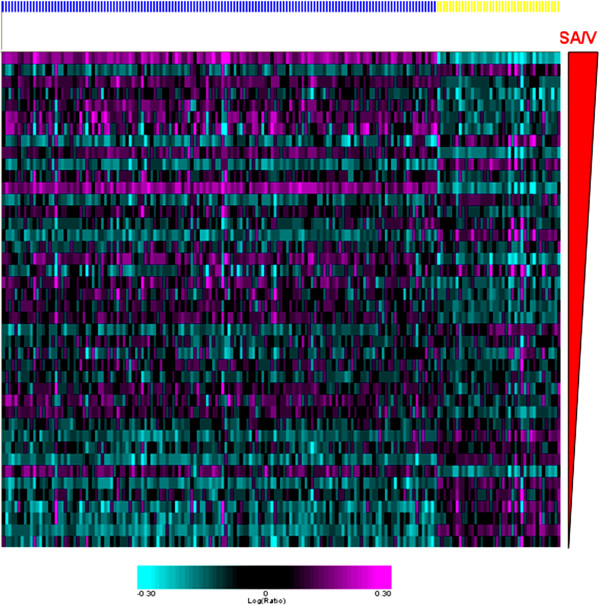
**A heat map shows the relationship between the level of expression of the 181 SA/V-correlated genes (columns) for all 42 of the samples in the discovery set.** The samples are shown in rows ordered by increasing SA/V. Genes whose expression was negatively related to SA/V are shown by the yellow bar while those whose expression was positively related to SA/V are indicated with the blue bar.

**Figure 2:**
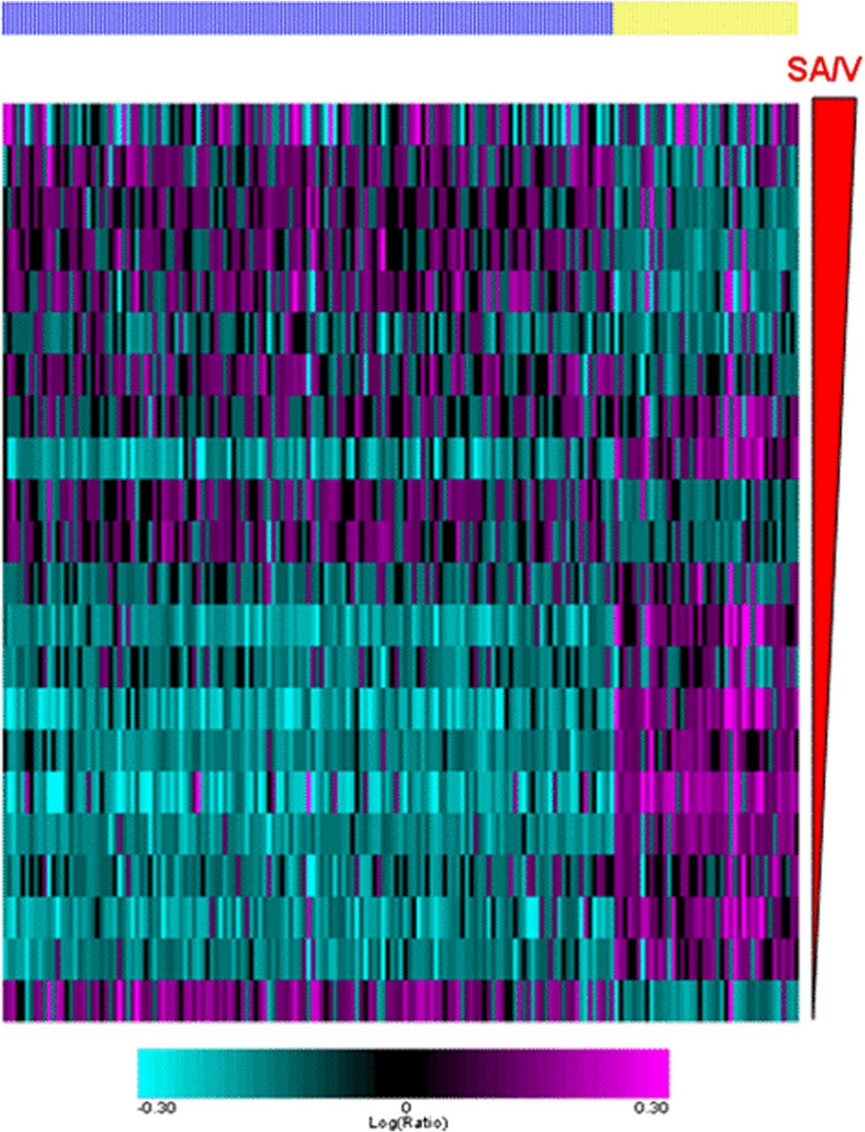
**A heat map shows the relationship between the level of expression of the 181 SA/V-correlated genes (columns) for the 22 samples in the replication set.** The samples are shown in rows ordered by increasing SA/V. Genes whose expression was negatively related to SA/V are shown by the yellow bar while those whose expression was positively related to SA/V are indicated with the blue bar. The pattern is similar to the discovery set.

The analysis identified 181 genes whose level of expression was related to SA/V in the discovery data set. Additional file 1: Table S4 lists these genes and the slope, intercept and p value for their relationship to SA/V. A visual appreciation of the expression of the 181 genes related to SA/V in the discovery set is shown in Figure 1. The data are arranged by SA/V. Although the data shown are the original data without model fitting, a SA/V-correlated trend is observed when all the samples are combined and sorted by increasing SA/V values. The level of expression of these transcripts was then related to the SA/V in the 22 samples which constitute the replication data set. Figure 2 shows that when the genes from the discovery set are arranged in the same order, the SA/V-correlated trend is preserved in the replication set.

**Figure 3:**
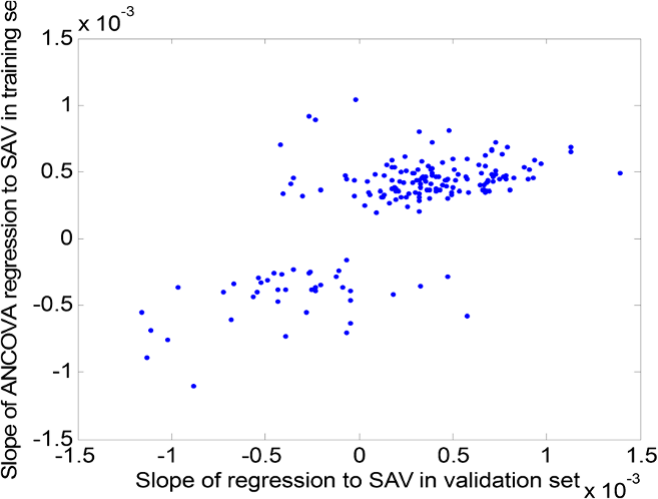
**Slopes of regression for 181 selected genes are compared between the discovery and replication sets.** The comparison shows a good concordance in the findings.

**Figure 4:**
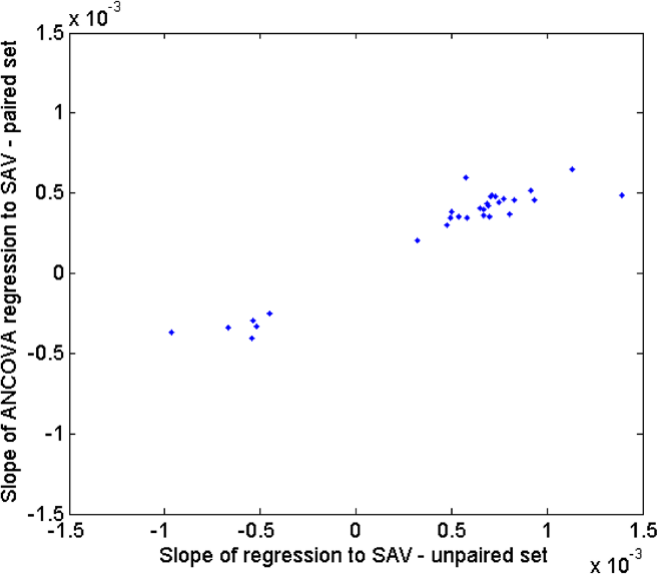
**Slopes of regression for the 30 genes that replicated at p < 0.05 in the replication data set.** The slopes for the replication data are on the x axis and those from the discovery set are on the y axis.

The slopes relating gene expression to SA/V from the discovery and the replication sets are compared in Figure 3 which shows that the slopes and direction of effect obtained from both discovery and replication sets are consistent, with an overall correlation of 0.6. If we use a slope p-value cutoff <0.05 in the replication set as a criteria to indicate replication of the initial relationship, 30 of the 181 genes achieve this level of significance (Table 2). Figure 4 is identical to Figure 3 except that it only shows the 30 genes that replicated based on the p value cut off in the replication sample set. These genes represent potential markers and candidates for subsequent follow up.

**Table 2:** **List of 30 replicated emphysema genes**

mRNA	Gene symbol	Gene name	Discovery slope	Discovery slope P value*	Replication slope	Replication slope P value
NM_005706	TSSC4	Tumor suppressing subtransferable candidate 4	-0.00037	0.039	-0.00096	0.003
AB067498	Esco1	Establishment of cohesion 1 homolog 1 (S. cerevisiae)	0.00038	0.018	0.00045	0.005
NM_018559	kiaa1704	KIAA1704	0.00040	0.023	0.00065	0.006
NM_004897	MINPP1	Multiple inositol polyphosphate histidine phosphatase, 1	0.00035	0.033	0.00070	0.006
Contig55580_RC			0.00049	0.029	0.00139	0.006
NM_012180	FBXO8	F-box protein 8	0.00040	0.010	0.00067	0.014
NM_153044	MORC2-AS1	MORC2 antisense RNA 1	-0.00040	0.022	-0.00054	0.015
NM_022876	SMN2	Survival of motor neuron 2, centromeric	0.00046	0.019	0.00083	0.017
NM_003838	FPGT	Fucose-1-phosphate guanylyltransferase	0.00035	0.031	0.00054	0.018
AF055030	PHF10	PHD finger protein 10	0.00036	0.011	0.00067	0.019
NM_007342	NUPL2	Nucleoporin like 2	0.00037	0.047	0.00081	0.019
Contig45624_RC	CBLL1	Cbl proto-oncogene-like 1, E3 ubiquitin protein ligase	0.00052	0.018	0.00091	0.020
NM_002103	GYS1	Glycogen synthase 1 (muscle)	-0.00033	0.017	-0.00052	0.020
AL359938	MEIS3	Meis homeobox 3; Meis homeobox 3 pseudogene 2	-0.00029	0.040	-0.00054	0.022
NM_014771	RNF40	Ring finger protein 40	-0.00034	0.027	-0.00067	0.024
NM_022877	SMN2	Survival of motor neuron 2, centromeric	0.00046	0.018	0.00077	0.024
Contig41498_RC	PTPN4	Protein tyrosine phosphatase, non-receptor type 4	0.00035	0.027	0.00049	0.024
NM_003084	SNAPC3	Small nuclear RNA activating complex, polypeptide 3	0.00044	0.015	0.00075	0.025
NM_013234	EIF3K	Eukaryotic translation initiation factor 3, subunit K	-0.00025	0.044	-0.00045	0.027
NM_006852	TLK2	Tousled-like kinase 2	0.00021	0.037	0.00032	0.028
X68560	SP3	Sp3 transcription factor	0.00065	0.012	0.00113	0.030
AY007149	CEP350	Centrosomal protein 350 kDa	0.00034	0.029	0.00058	0.030
Contig51940_RC	GABPA	GA binding protein transcription factor, alpha subunit 60 kDa	0.00046	0.035	0.00093	0.033
NM_032557	USP38	Ubiquitin specific peptidase 38	0.00048	0.017	0.00073	0.034
NM_015153	PHF3	PHD finger protein 3	0.00060	0.034	0.00057	0.036
NM_004162	RAB5A	RAB5A, member RAS oncogene family	0.00030	0.045	0.00048	0.037
NM_022875	SMN2	Survival of motor neuron 2, centromeric	0.0004843	0.015	0.00071	0.039
NM_005316	GTF2H1	General transcription factor IIH, polypeptide 1, 62 kDa	0.00042	0.010	0.00069	0.039
Contig53191_RC	GPD2	Glycerol-3-phosphate dehydrogenase 2 (mitochondrial)	0.00043	0.043	0.00069	0.041
NM_017411	SMN2	Survival of motor neuron 2, centromeric	0.00048	0.029	0.00071	0.044

*All FDR values were >0.1 in the discovery samples.

The results of the network and pathways analyses are shown in Table 3. Using MetaCore, the top significantly enriched pathways (Minimum P = 2.786E-05) were cell cycle regulation of G1/S transition, followed by the Immune response HMGB1/TLR signaling pathway (P = 6.224E-04). In terms of disease processes, “Starvation” ranked first (P = 1.042E-04). Gene Ontology (GO) processes’ enrichment presented in Additional file 1: Table S5 identified processes related to cellular macromolecule metabolism (P = 1.417E-10), and ubiquitination (P = 2.103E-08) pathways as being significantly enriched. Many of these genes are related to the process of ubiquitination or deubiquitination.

**Table 3:** **MetaCore pathway and network analysis results for 181 genes related to emphysema**

**Pathway analysis**		
**#**	**Maps**	**p-value**
1	Cell cycle_Regulation of G1/S transition*	2.786E-05
2	Cell cycle_Role of SCF complex in cell cycle regulation*	3.259E-04
3	Cell cycle_ESR1 regulation of G1/S transition*	4.803E-04
4	Immune response_ HMGB1/TLR signaling pathway*	6.224E-04
5	Immune response_HSP60 and HSP70/TLR signaling pathway	2.039E-03
**Diseases by Biomarker**		
**#**	**Diseases**	**p-value**
1	Starvation*	1.042E-04
2	Cystadenocarcinoma, Serous	1.066E-03
3	Cystadenocarcinoma	1.328E-03
4	Neoplasms, Cystic, Mucinous, and Serous	1.401E-03
5	Neoplasms, Complex and Mixed	1.497E-03
6	Motor Neuron Disease	3.125E-03
**Process Networks**		
**#**	**Networks**	**p-value**
1	Proteolysis_Proteolysis in cell cycle and apoptosis*	7.634E-04
2	Cell cycle_Mitosis*	9.314E-04
3	Cell cycle_S phase	1.896E-03
4	Transcription_mRNA processing	2.716E-03
5	Transcription_Chromatin modification	5.222E-03
6	Signal Transduction_BMP and GDF signaling	8.269E-03
7	Cell cycle_G1-S	1.407E-02
8	Cytoskeleton_Spindle microtubules	1.531E-02
9	Signal transduction_NOTCH signaling	1.720E-02
10	Cell cycle_G2-M	3.453E-02

*Donates that the enrichment for pathways, diseases and networks is significant at FDR <0.1.

Interestingly, analysis of the 30 replicated genes showed significant enrichment in disease biomarkers for muscular atrophy (p = 4.239E-04) and proteostasis (p = 4.829E-05).

### Protein replication

**Figure 5:**
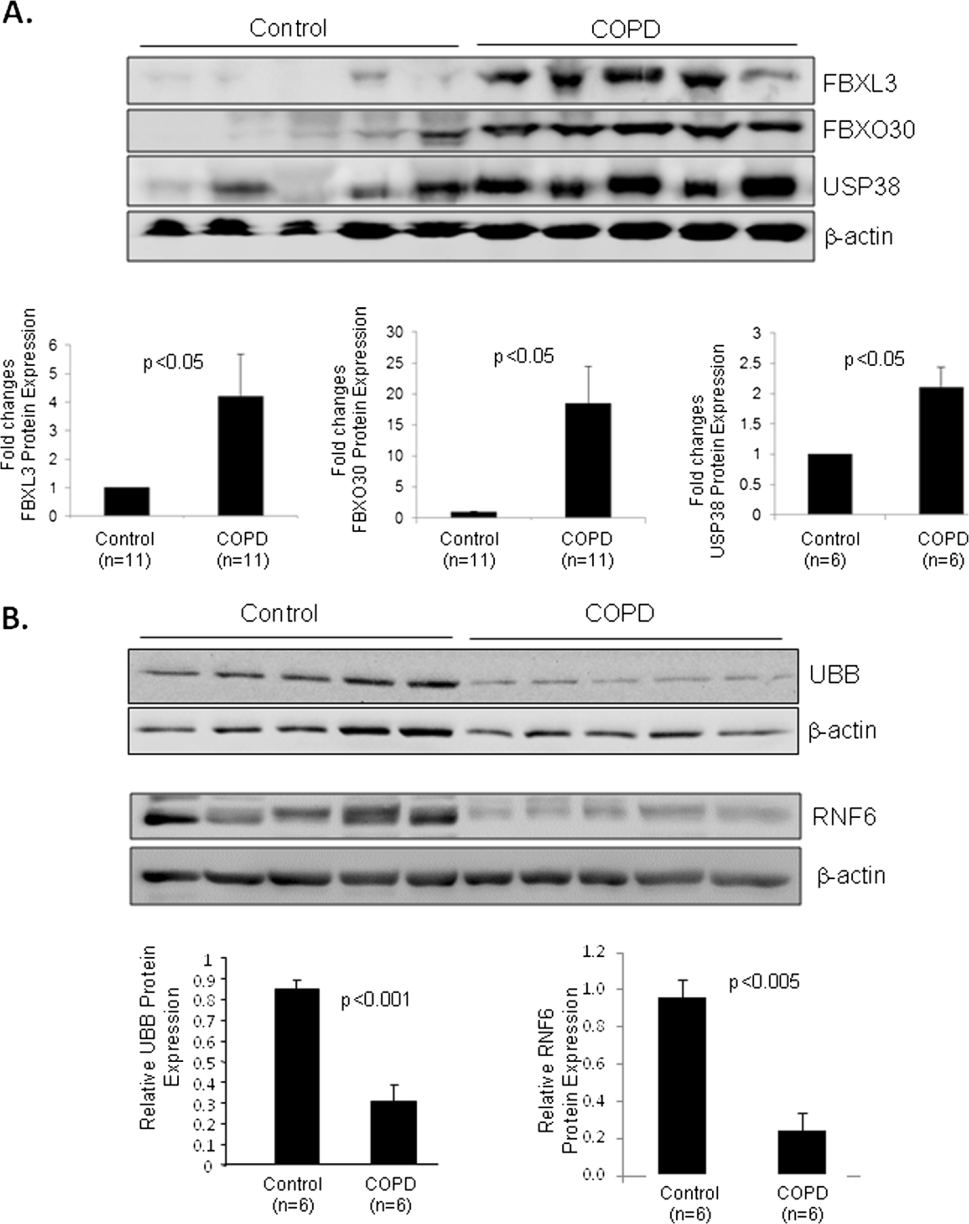
**Protein expression of ubiquitination genes in lung tissues from control and COPD patients.** Lung homogenates were prepared and Western blotting was performed to examine protein levels with the antibodies specified. β-actin was probed as a protein loading control. Protein levels were quantified by densitometric analysis wit the NIH ImageJ program and normalized to β-actin expression. **A**: results for FBXL3, FBXO30, and USP38 showing upregulation in COPD. **B**: results for UBB and RNF6 showing downregulation in COPD. Data are means ± SE, and significance was determined by Students’ t-tests.

The protein level of five of the 9 ubiquitination-associated genes (FBXL3, FBXO30, USP38, UBB, and RNF6) was significantly different between control and COPD lung tissue by western analysis (Figure 5). FBXL3, FBXO30, and USP38 were upregulated at the protein level (Figure 5A), which is opposite in direction to the changes in mRNA. This could be a reflection of increased protein stability. However UBB and RNF6 protein were downregulated consistent with the gene expression profile (Figure 5B). The protein abundance of the remaining four genes (UBE4A, RNF184, TBLR1, and UHRF2) was not significantly altered (data not shown).

## Discussion

Most studies of gene expression in the lungs of patients who have COPD have compared tissue from subjects with and without COPD based on lung function [3, 5–7].

Although COPD is defined by abnormalities of lung function there is convincing evidence that there are two pathological processes that contribute to the functional impairment [8]; loss of lung elasticity characterized pathologically by emphysema and inflammatory narrowing and obliteration of the small conducting airways [9]. We used a morphometric measure of emphysema, derived from the same sample of lung tissue that was used for transcriptomic analysis, as a continuous variable to relate gene expression to structural changes.

The design of the discovery study using a pair of lung samples from each individual, one with the highest and one with the lowest SA/V ratios, allowed us to account for patient-to-patient variability. An advantage of this design is that the demographic distribution of patients is of much lesser concern than for between- subject comparisons, where serious attention must be given to stratification and balancing of patients with respect to potentially confounding demographic attributes. Transcripts that significantly correlated with SA/V ratio after adjusting for patient gene expression variation constitute relevant biological candidates.

Although most studies of transcriptional profiles of COPD patients have focused on expression changes associated with disease status or level of lung function, Campbell et al. recently reported the results of a study examining gene expression as related to alterations in local lung architecture [10]. This study examined the correlation of gene expression with Lm (Lm = mean linear intercept, a microscopic measure of emphysema directly related to lung SA/V). Despite the apparent similarity in the design of Campbell et al. and our study, we found no genes in common. While this is undoubtedly due, at least in part, to differences in patient populations, the specifics of sample preparation and/or the expression platform (Agilent versus Affymetrix), another factor may have been experimental design. Two of the eight lungs in the study of Campbell et al. were from normal individuals, and these had substantially lower Lm than in COPD. This creates a potential bias such that many genes reported as correlating with Lm were also dependent on COPD status. While this covariate was technically accounted for with the inclusion of a random patient effect in the fitted mixed models, the authors did not remove genes that had a significant patient effect as we did. Indeed, 78 of 126 genes reported by Campbell et al. were differentially expressed in normal donors compared to COPD patients (p-value <0.001 by student’s T-Test) and 65 of these genes also had a significant patient effect in our study (p-value of alpha <0.05). Thus, differences in results may indicate that the results of Campbell et al. reflect a mix of gene expression changes due to emphysema and disease status.

The most significantly enriched pathways we identified relate to the regulation of G1/S transition, and to high mobility group box protein 1/toll like receptor (HMGB1/TLR) signaling. Further discussion of the potential role of these genes is included in the online supplement.

Many of the genes identified as being related to SA/V are involved in the handling of proteins. Cellular protein homeostasis, also known as proteostasis, involves the control of the conformation, concentration, binding interactions, and localization of individual proteins within and outside the cell. The proteostasis network refers to the >2000 genes in mammals encoding proteins that work together as a system to control protein concentration and conformation through interactions of the proteome with chaperone systems and folding enzymes as well as via protein degradation mediated by the ubiquitin proteasome system [11].

There is evidence that an imbalance in proteostasis contributes to certain diseases [12]. Our data implicating genes involved in the handling of protein suggests that an imbalance in the synthesis and degradation of protein may be involved in the genesis of emphysema. This idea is not new; Min et al. used immunohistchemistry to examine the expression of proteins involved in protein processing and apoptosis in the lungs of individuals with varying severity of COPD. They show accumulation of poly-ubiquitinated proteins in insoluble aggregates in the lungs of subjects with emphysema [13]. Cantin and Richter also suggested that proteostasis imbalance is important in obstructive lung diseases [14].

In particular, genes involved in the ubiquitin-proteosome pathway were significantly over-represented in the present study. The ubiquitin-proteasome system (UPS) serves a crucial function in protein quality control to maintain cellular proteostasis through the degradation of misfolded/damaged proteins and the turnover of normal short-lived regulatory proteins. Recently, impairment of the UPS has been reported in several lung diseases, including COPD/emphysema and pulmonary fibrosis [15, 16]. It was shown that lung tissue from COPD patients with severe emphysema has aberrant accumulation of ubiquitinated proteins [13], a phenomenon commonly observed in protein conformational diseases, including neurodegenerative and heart diseases [17, 18].

Increased abundance of ubiquitinated conjugates could be the result of decreased proteasome activity and/or increased protein ubiquitination. Upon exposure to cigarette smoke, the proteolytic activities of the proteasome in human alveolar epithelial cells and mouse lung were markedly decreased [19, 20]. We also demonstrated a consistent reduction of all three proteasome activities (chymotrypsin-, trypsin-, and caspase-like) in human COPD lung tissues as compared to control lung (Additional file 2: Figure S3), although the latter two changes did not reach statistical significance probably due to small sample size.

In addition to disposal of misfolded/damaged proteins, the UPS also plays a key role in the regulation of many fundamental cellular functions, including apoptosis, cell cycle regulation, antigen processing, and transcriptional regulation via controlling the degradation of normal regulatory proteins [21–23]. Both FBXL3 and FBXO30 are F-box proteins, critical components of the SCF (SKP1-cullin-F-box) E3 ligases which are involved in the degradation of signal and cell cycle proteins [24]. Upregulation of these proteins suggest a role for apoptosis and cell cycle arrest in the pathogenesis of emphysema. USP38 is a deubiquitinating enzyme and was recently identified as a susceptibility gene for asthma [25]. Although the exact targets of USP38 remain unclear, it is speculated that upregulation of USP may reflect a cellular response to inflammation and injury. The gene UBB encodes polyubiquitin precursor protein which is processed by deubiquitinating enzymes to produce a single ubiquitin moiety and ribosomal proteins [26]. Down-regulation of UBB will lead to decreased availability of the free ubiquitin pool, thereby resulting in reduced protein ubiquitination and subsequent degradation. RNF6 is a RING (Really Interesting New Gene) finger domain E3 ligase and LIM kinase 1 (LIMK1) has been identified as a substrate of RNF6 [27]. LIMK1, a serine/threonine kinase involved in the regulation of actin polymerization and microtubule disassembly, was shown to promote the disruption of endothelial barrier and inflammatory infiltration in mouse lung [28]. It is therefore plausible to assume that downregulation of RNF6 plays a role in the chronic inflammation of COPD.

**Figure 6:**
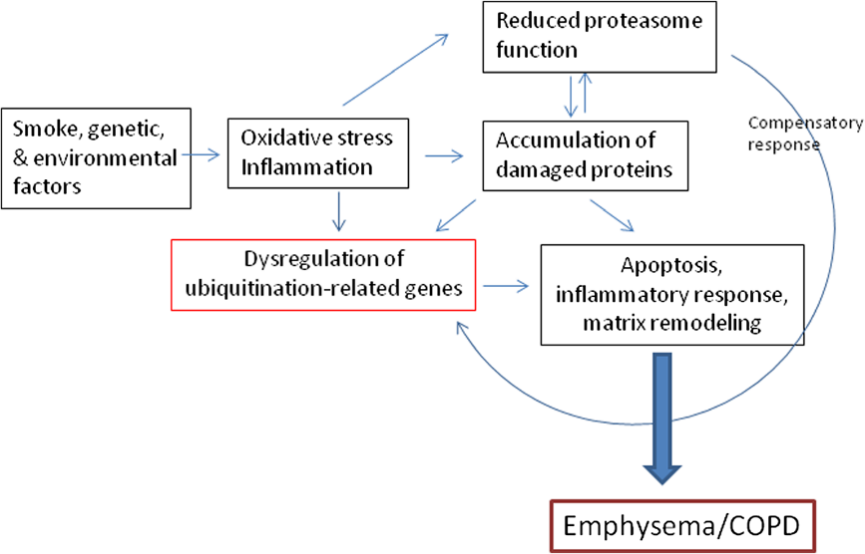
**Proposed model of dysregulation of the ubiquitin-proteasome system leading to the pathogenesis and progression of emphysema/COPD.** Oxidative stress and inflammation induced by smoke, genetic or environmental insults result in dysregulation of ubiquitination-related genes and impairment of the proteasome function. Accumulation of abnormal proteins in the lung as a result of increased production and decreased degradation causes further damage of the proteasome function and dysregulation of UPS-related genes. Aberrant regulation of the UPS results in apoptosis, inflammation, and matrix remodeling, pathogenic characteristics of emphysema/COPD. Damaged proteasome function can also cause compensatory upregulation of genes associated with the UPS.

Figure 6 summarizes the proposed mechanisms by which smoke and ROS-mediated modulation of the UPS could contribute to the pathogenesis and progression of COPD by participating in the regulation of apoptosis, the inflammatory response, and interstitial fibrosis. Future studies are required to address the function and regulation of this system in COPD/emphysema and to further explore the specific mechanisms involved.

There are several limitations to this study. The sample size is relatively small and thus the possibility of false negative or positive associations is real. We addressed this, in part, by using one portion of the samples as a derivation set and one portion as a replication set. Figure 3 shows that the level of expression of 164 of the 181 genes were associated with SA/V in the same direction in the replication set as in the derivation set and 30 of the 181 genes showed significant (p < 0.05) association with SA/V in the replication set (with the same direction of effect – see Figure 4). We used all 181 genes, not only the 30 that replicated, to examine for enriched networks using MetaCore®. We feel we are justified in this approach given that the vast majority of expression changes were in the same direction. Another limitation is the fact that samples were done in two batches which had a substantial systematic effect on gene expression. Although we adjusted for this bias statistically, it is possible that some true positive associations could have been missed due to this adjustment.

Finally, changes in gene expression do not confirm that these genes are causal to emphysema. Some of the changes could be a consequence of the molecular processes that result from the development of emphysema.

## Conclusions

The analysis approach adopted in this study allowed examination of the relationship between gene expression and SA/V, a measure of tissue destruction by emphysema. Gene set annotation and pathway analyses implicated genes involved in protein ubiquitination and the proteasome pathways and protein analysis confirmed the abnormal accumulation of protein-ubiquitin conjugates and the altered expression of genes involved in protein ubiquitination in the lungs of obstructed smokers.

### Availability of supporting data

Additional data is available in a supplementary material document. The microarray data was deposited on Gene Expression Omnibus (GEO; http://www.ncbi.nlm.nih.gov/projects/geo/). The GEO accession number is GSE63073.

## Electronic supplementary material

Additional file 1: **Online supplementary contacting detailed methods, additional results and discussions.** (DOC 860 KB)

Additional file 2: Figure S3: Proteasome activities in lung tissues from control and COPD patients. Lung homogenates were prepared and chymotrypsin-, trypsin-, and caspase-like proteasome activities were measured as described in the Materials and Methods. Results are expressed as means ± SE. Significance was determined by Student's *t*-test. (TIFF 71 KB)
