# Supplementary material for: Genes related to emphysema are enriched for ubiquitination pathways
Source: BMC Pulm Med. 2014 Nov 29;14:187. doi: 10.1186/1471-2466-14-187 (PMC4280711; doi:10.1186/1471-2466-14-187)
Supplement: Supplementary file 1 — Additional file 1: Online supplementary contacting detailed methods, additional results and discussions. (DOC 860 KB) [file 12890_2014_636_MOESM1_ESM.doc]

**Genes related to emphysema are enriched for ubiquitination pathways**

**Additional Material**

Sergey Stepaniants^1ψ^,I-Ming Wang^2ψ^, Yves Boie^2^, James Mortimer^2^, Brian Kennedy^2^, Mark Elliott^3^, Shizu Hayashi^3^, Honglin Luo^3^, Jerry Wong^3^, Leanna Loy^3^, Silvija Coulter^2^, Jennifer Harris^2^, Christopher J Roberts^2^, James C Hogg^3^, Don D Sin^3^, Gary O’Neill^2^, Michael Crackower^2^ , Melody Morris^2^., Peter D Paré^3^ and Ma’en Obeidat ^3^

^1^Covance Genomics Laboratory, LLC, ^2^Merck Research Laboratory, ^3^UBC Centre for Heart and Lung Innovation, St Paul’s Hospital

ψ Sergey Stepaniants and I-Ming Wang contributed equally to this study

Correspondence to Dr Peter D Paré (Rm 166, Centre for Heart Lung Innovation, St. Paul’s Hospital, 1081 Burrard St., Vancouver, BC, Canada V6Z 1Y6 Tel:604-806-8346, Fax:604-806-8351, email: [Peter.pare@hli.ubc.ca](mailto:Peter.pare@hli.ubc.ca))

Supported by the Canadian Institute for Health Research and Merck Frosst Canada

**METHODS:**

**Subject selection and Experimental design:**  184 subjects had their resected lung tissue frozen and archived in a biobank in a manner suitable for gene expression studies and provided informed consent for their tissue to be used for COPD research using methods approved by the Providence Health Care Clinical Ethics Review Board. From these 184 subjects we selected 74 subjects to represent a range of smoking and lung function. We randomly selected 6 of the 12 non-smokers in the biobank and randomly sampled ~1/3 of those in the lower GOLD categories (GOLD 0-1), ~1/2 of those in GOLD 2, and all 3 of the subjects in GOLD 3. Of these, 43 were found to have mRNA of suitable quality for gene expression profiling. For 21 of these subjects an additional sample of lung from a region with a different SA/V was also profiled.

The subjects whose RNA did not pass quality control criteria did not differ from those included in the study with respect to age (63.4±2 versus 62.8±2 years); male/female ratio (15/11 versus 27/16); FEV1% predicted (86.8±4 versus 86.5±3) or pack years smoked (34.4±6.3 versus 39.1 ±3.2 ). In addition there was no difference in the time from tissue collection to RNA extraction for those samples which yielded high or lesser quality RNA.

Gene expression was tested in two phases. In phase 1, one sample from each of the 43 subjects was subjected to Agilent gene expression profiling as described in a previously published manuscript . In phase 2 one or two additional samples were selected from 40 of the subjects and SA/V was measured for comparison with the SA/V value obtained on the initial sample. From these 40 samples, 21 additional samples were selected for profiling. These were selected as the samples which showed the largest difference in SA/V from the initial sample (higher or lower SA/V). Thus for 21 individuals we had two samples which were discordant for emphysema (SA/V) and we used these paired samples as a derivation set to identify transcripts whose level of expression was related to SA/V. Table E1a and E1b show the lung function and SA/V ratio for each of the individuals in the derivation and replication sample.

**Lung Function Measurement:** Prior to surgery subdivisions of lung volume, spirometry and single breath diffusing capacity were measured as previously described and according to ATS standards (10). A modified ATS questionnaire was applied to gather demographic and clinical information. A detailed smoking exposure was determined and expressed as pack years. Based on smoking history and lung function, subjects were classified as lifetime non-smokers or into the GOLD categories of COPD severity (3). We expressed forced expiratory flow in one second as percent of predicted (FEV1%P) and as a percentage of the forced vital capacity (FEV1/FVC%). DLCO was expressed as a percent predicted Table 1 shows the number of patients in each GOLD category and their mean smoking history and lung function.

Prior to surgery subdivisions of lung volume, spirometry and single breath diffusing capacity were measured as previously described and according to ATS standards (10). A modified ATS questionnaire was applied to gather demographic and clinical information. A detailed smoking exposure was determined and expressed as pack years. Based on smoking history and lung function, subjects were classified as lifetime non-smokers or into the GOLD categories of COPD severity (3). We expressed forced expiratory flow in one second as percent of predicted (FEV1%P) (ref) and as a percentage of the forced vital capacity (FEV1/FVC%). DLCO was expressed as a percent predicted. (ref) Table 1 shows the number of patients in each GOLD category and their mean smoking history and lung function.

**Tissue processing**: Immediately following resection, the lung or lobe was obtained from the operating room and after the clinical specimens of the lesion, lymph nodes and the resection margin were obtained the lobes and lungs were inflated using a 50% mixture of Cryomatrix^R^ and saline and frozen in liquid nitrogen fumes. The frozen lungs and lobes were then cut into 7-15 two cm thick slices using a band saw and multiple randomly stratified cores of frozen lung were acquired (1-3/slice) using a power driven hole saw fitted with a 1.5cm diameter bit (11). –Cores were frozen at 80^o^ C for later cryosectioning and RNA extraction. Three cores from each subject were randomly selected.

Frozen sections were obtained from the surface of the ½ core immediately adjacent to the portion to be used for RNA extraction. The 10 micron sections were stained with hematoxylin and eosin and digital images of the entire sample were captured using a Nikon Eclipse E600 microscope fitted with a SPOT camera. The severity of emphysema in each core was determined by analyzing 6 random fields/slide at 24x magnification and calculating the lung surface area to volume ratio (SA/V) using an in-house point counting program. This program counts tissue endpoints, air endpoints and intercepts and calculates the surface/volume ratio using the following equations:

Volume Fraction of Tissue (Vv tis) = tissue endpoints / total endpoints

Surface density (Sv)= (4 x Intercepts) / (Grid Length x tissue endpoints)

Surface Area / Volume Ratio (SA/Vol)= Sv x Vv tis

The terms endpoints and intercepts refer to the morphometric program for calculating mean linear intercept and the SA/V ratio. Each line in the grid that is superimposed on the image of the lung parenchyma has two ends and one counts the number of ends that are on alveolar walls and the number on air spaces. The intercepts are the number of times the lines traverse an alveolar wall.

The image analysis was performed using Image Pro Plus 4.0 (Media Cybernetics, Silver Spring, MD). The morphometric analysis was performed by an observer (LL) who was blinded to all information except that present on the slide.

**RNA processing and Quality Control:** Homogenization of the lung cores for RNA extraction was performed in 10 ml of Trizol reagent. Phase separation and RNA precipitation were performed according to reagent protocol. RNA pellets were dissolved in 100 ul of RNase-free water. The Qiagen RNeasy Mini Protocol for RNA cleanup was then followed for further RNA cleanup and on-column DNase digestion. Elution steps were performed twice to maximize RNA recovery.

Assessment of sample concentration and integrity are essential for ensuring the quality of expression data. A fixed volume (5 µL total) of each total RNA sample was used to determine both sample concentration and integrity. Samples concentrations were assayed by determining the OD260 by UV spectrophotometer, with a passing concentration range set at >0.11 ug/ul. Samples whose concentrations exceed the upper limits of the amplification protocol were diluted to a target concentration of 0.2 ug/ul and re-assayed to confirm accurate dilution. RNA sample integrity was evaluated by calculating the rRNA ratio of 28S/18S using the Agilent Bioanalyzer capillary electrophoresis system. The passing criteria for use in RNA microarray experimentation was a 28S/18S rRNA ratio between 0.75 - 3.02. Samples passing RNA sample QC were released for subsequent amplification and hybridization.

As an array is scanned in the Agilent DNA Microarray Scanner carousel, a TIFF image is produced revealing the fluorscence from the Cy3 (570 nm) and the Cy5 (670 nm) channels. Several quality metrics were used to assess the quality of the microarray and the quality of the RNA. These metrics provide guidance into in probable causes of failure and likelihood of correction upon repeat. Potential failures include inefficiencies in amplification or subsequent processing, or physical defects on the array features. Profile QC metrics are based on numerous criteria including co-amplified synthetic spike-ins, full array summary statistics, array manufacturing assessment gridline spike-ins, and individual feature assessment. All QC metrics were evaluated through an automated process and displayed in Resolver. By default, metrics which indicate defective arrays or array processing were automatically recoupled, whereby a second aliquot of the amplified cRNA samples was coupled to Cy dyes, then combined and hybridized to a new array.

cRNA Labeling and Expression Profiling. cDNA was produced from 5 μg total RNA by reverse transcription (RT) using Moloney murine leukemia virus (MMLV) RTase and then transcribed into cRNA by in vitro transcription (IVT) using T7 RNA polymerase. 5-(3-Aminoallyl)uridine 5V-triphosphate (Sigma) was incorporated into cRNA in the IVT reaction. For cRNA labeling, the allylamine-derivatized cRNA products were reacted with N-hydroxysuccinimide esters of Cy3 or Cy5 dyes (Amersham Pharmacia Biotech, Piscataway, NJ) as described previously (E 2). The resulting labeled probes were hybridized to hu25k oligonucleotide microarrays. All hybridizations were done in duplicate with fluor reversal on two microarrays to compensate for potential biases due to the different chemical properties of Cy3 and Cy5 dyes. The arrays were scanned to detect the level of gene expression for 21,000 genes as described previously (E3). Fluorescence intensities of scanned images were quantified, normalized and corrected to yield the transcript abundance of a gene as an intensity ratio with respect to that of the signal of the reference pool.

**Microarray study design and methodology:** RNA from 8 GOLD 0 subjects (non-obstructed smokers) was pooled to form the reference RNA. For 2-color array experiment, a reference pool with sufficient RNA to compare with each individual sample within the study (including samples used as the reference pool) is necessary. This pool forms the “control”. The amount of RNA from each sample to be included in the reference pool needs to be the same to keep this pool balanced. We chose smokers who did not develop airflow obstruction (GOLD 0) as our “control”. We tried to include as many GOLD 0 samples in the pool as possible and the 8 samples chosen are those on whom we had sufficient RNA to contribute to the pool and still have enough RNA to be compared back to the pool.

The reverse transcribed cDNA from this reference was used for competitive hybridization against all of the other samples including all of the GOLD 0 samples which made up the reference pool. Microarray profiling was done as previously described (E-4). The two-colour microarrays were scanned using the Agilent scanner and proprietary image acquisition software.  Rigorous image QC using proprietary software was performed. The microarray data was deposited on Gene Expression Omnibus ((GEO; http://www.ncbi.nlm.nih.gov/projects/geo/ )). The GEO accession number is GSE63073.Experimental QC was performed in MATLAB (Mathworks, Inc. Natick, MA, [http://www.mathworks.com](http://www.mathworks.com/)). At this point, spiked-in exogenous mRNA hybridizations were examined for a large number of known problems and attempts were made to explain any abnormal trends or outlier arrays. Expression data were loaded into the Resolver (Rosetta proprietary software database for transformation,, normalization and error modeling (<http://www.ceibasolutions.com/rosetta-about>) . Fluor-reversed pairs for each sample were combined to give a single log-ratio and a p-value for technical variability for each biological sample compared to its appropriate control. Next, 1D and 2D clustering and classifier analysis was used to get an overview of the experiment. Data mining of these clusters was performed using prior biological knowledge, known pathways, and gene ontology or keyword over-representation. Data was exported to Spotfire/decisionsite for further analysis and visualization (Spotfire, Somerville MA, [http://spotfire.com](http://spotfire.com/)).

**Pathways, Gene ontology (GO) and Disease set enrichment analysis of 181 genes :** We used MetaCore from the GeneGo package to identify pathways, diseases and GO processes overrepresented in the 181 emphysema-related genes. Gene symbols were used as input for MetaCore analysis. MetaCore maps these gene IDs onto gene IDs in both a proprietary in- house and the GO public ontologies represented in MetaCore pathway maps and networks. Mapping procedure involves calculating P values and false discovery rate (FDR) adjusted P values of the matches found. The GO categories or disease sets with a FDR corrected P value <0.05 were considered significant.

**Batch Effect:** The phase 1 and 2 gene expression data sets (batches) correspond to the paired patient samples with the different SA/V ratios and the replication set in which one sample was derived from each individual. Since phase 1 was performed earlier than phase 2, different amplification protocols and reference pools were used which created a significant difference shown in Figure S1. When one-way ANOVA analysis was used to assess the significance of this potential batch effect for each gene, it resulted in ~ 14,000 genes significantly different between the phases with p < 0.01. Therefore, it is hard to imagine that this overwhelming difference can be triggered by the SA/V differences alone, and not be impacted also by a technical artifact between the phase 1 and 2 data. Since this systematic bias between the phase 1 and 2 data could confound relationships between gene expression and SA/V ratios adjusting for this systematic bias will reduce both the number of false and true positive findings. We choose to do such an adjustment in order to minimize the spurious findings.

A simple way to adjust for this systematic difference is to center each data set about its mean. To accomplish this, the arithmetic mean of logarithms of gene expression ratios for each gene was computed across patients in each phase. These corresponding mean values were then subtracted from the log ratio of each gene in phase 1 and 2 data respectively. This effectively removes the systematic difference between the phases. An indication that this adjustment/preprocessing step improves the data is provided by the co-clustering of the originally significantly different pairs of samples from the 21 patients whose samples were profiled in phase 1 and 2 (Figure S2). In addition, this co-clustering is driven by a large number of genes, which indicates that the patient effect is one of the dominant effects in the study. This is not surprising, since variation of baseline gene expression between humans is large due to genetic and environmental factors. This significant patient effect is adequately accounted for by our paired experimental design and the corresponding statistical model.

**Western Blotting:** Frozen human lung samples were ground into fine powder using the liquid nitrogen cooled mini mortar and pestle system (Sigma) and suspended in lysis buffer (50 mM Tris-HCl, pH 7.2, 250 mM NaCl, 0.1% NP-40, 2 mM EDTA, 10% glycerol) containing protease inhibitor cocktail (Roche). Samples were centrifuged at 14,000 rpm for 10 min to remove insoluble materials. Protein concentration was determined by Bradford assay (BioRad). Equal amounts of protein were subjected to sodium dodecyl sulfate-polyacrylamide gel electrophoresis and then transferred onto nitrocellulose membranes (GE healthcare). The membranes were blocked for 1 h with 5% nonfat dry milk solution containing 0.1% Tween 20. The blots were then incubated for 1 h with the primary antibody, followed by incubation for another hour with a secondary antibody. Immunoreactive bands were visualized by enhanced chemiluminescence (GE healthcare) and captured by ChemiGenius Bioimaging System (Syngene). The target bands were quantitated by densitometric analysis with the NIH ImageJ program. The monoclonal anti-β-actin antibody was purchased from Sigma. The antibodies against UBB, FBXO30, FBXL3, and RNF184 were from Abnova. The antibodies against RNF6, UBE4A, and USP38 were from Abcam. Anti-UHRF2 and Anti-TBLR1 antibodies were obtained from Aviva System Biology and Bethyl Laboratory, respectively.

**Proteasome activity assay:** Lung sample homogenates freshly prepared as described above, but in the absence of protease inhibitors, were used to measure proteasome activity as previously described . Briefly, 10 μg of lung homogenates was added to an assay buffer (20 mM Tris-HCl [pH 8.0], 1 mM ATP, and 2 mM MgCl_2_) in the presence of 75 μM synthetic fluorogenic substrate to a final volume of 100 μl. The fluorogenic substrates, Bz-Val-Gly-Arg-AMC (Biomol), Suc-Leu-Leu-Val-Tyr-AMC (Calbiochem), and Z-Leu-Leu-Glu-AMC (Calbiochem), were used to determine the 20S proteasome trypsin-like, chymotrypsin-like, and caspase-like activities, respectively. The mixtures were incubated at 30°C for 1 h, and the fluorescence product AMC in the supernatant was measured at a 465-nm emission wavelength using a fluorometer.

**DISSCUSSION:**

Our experimental design differed from most previous studies of gene expression in COPD since we profiled two tissue samples from each of the subjects in the discovery cohort and validated emphysema related signals in a replication cohort. The design of the discovery set using the pair of lung samples with highest and lowest SA/V ratios from the same individual allowed us to account for patient-to-patient variability.. While none of our findings passed FDR < 0.1 cutoff, indicating the absence of “low hanging fruits”, we nevertheless decided to pursue subsequent analysis and benefit from the availability of a replication set. It is not uncommon for genomic experiments to artificially dilute potential real findings by the presence of a large number of probe sets involved in the analysis. It is possible to imagine a situation that a real signal could be buried in an arbitrarily large number of small signals. While adjusting for multiple hypothesis testing is an important and conservative way to cut down on false findings, the replication of findings in an independent set is an essential step which constitutes the ultimate check for reproducibility. Clearly, it would have been advantageous if the replication set consisted of paired patient samples as well. However, this design was driven by the limitations of sample availability. The fact that the statistical analysis was performed on the paired samples and the replication set consists of singleton samples poses certain challenges, as it is impossible to evaluate and adjust for the patient effect in the replication set. This is why only those genes with a small patient effect (patient p-value > 0.1) in the discovery set were selected for the replication. It is possible, that the other genes significantly associated with SA/V could be as good or even a better choice for the subsequent follow up. However a paired replication set will be required to test that hypothesis.

**HMGB1:** HMGB1 is present in the nucleus where it is loosely bound to chromatin , and outside the nucleus it is bound to the receptor for advanced glycation end products (RAGE) and functions as a potent mediator of inflammation and cell migration . RAGE was recently identified in a number of genome-wide association studies (GWAS) to be associated with lung function measures . HMGB1 also signals through TLR2 and TLR4 . TLRs play an important role in innate immunity system, and in the acute and chronic lung inflammation

**Ubiquitin-proteasome system, starvation, BMI and muscle wasting in COPD:** The network and pathway analysis done on the SA/V-related gene expression pattern was related to “Starvation”. Besides the evidence of emphysema in the Warsaw Ghetto and in animal models of starvation (discussed in main paper) there are additional evidence linking the processes.

Coxson et al. have shown suggestive evidence of emphysema in patients who have anorexia nervosa . They used a quantitative CT method and found that CT lung density was significantly lower in anorexic patients than in the age-matched control group. They also reported significant relationships between BMI and the CT estimate of “emphysema” and between the extent of low attenuation areas and diffusing capacity.

The relationship between COPD and cachexia is well established and there is accumulating evidence that the cachexia is associated more specifically with the emphysema sub-phenotype of COPD. Ogawa et al found that body mass index (BMI) was significantly lower in smokers with emphysema-predominant COPD as opposed to smokers with airway-dominant COPD despite similar degrees of airway obstruction . They concluded that there was a relationship between the propensity to develop emphysema and to lose body mass but could only speculate regarding whether emphysema predisposes to weight loss or weight loss predisposes to emphysema. Kurosaki measured fat free body mass (FFM), smoking intensity, expiratory flow, diffusing capacity and the extent of emphysema in a group of 112 COPD patients. FFM was associated with smoking (pack years), lung function and emphysema but in a multivariate analysis only pack years and the extent of emphysema remained significant .

Although the mechanism of muscle wasting in COPD is not known it is thought to represent an imbalance between the processes of protein synthesis and protein breakdown. If there is an increase in cell apoptosis and/or a decrease in regenerative capacity atrophy will occur. It has been postulated that in COPD, muscle breakdown occurs via the activation of the adenosine triphosphate (ATP)-dependent ubiquitin–proteasome pathway coupled with reduced anabolic hormones . Interestingly, there is an increasing body of evidence which has demonstrated that the ubiquitin-proteasome system is also involved in the skeletal muscle and diaphragmatic abnormalities in COPD patients. . However, in contrast to its impaired function in lung, increased UPS activation is believed to be responsible for cachexia and diaphragm atropy observed in patients with severe COPD . Although the exact mechanism is still not clear, it has been proposed that systemic inflammation and production of ROS are the mediators leading to enhanced proteasome activity and/or increased expression of muscle-specific E3 ligases which target skeletal proteins for proteasomal degradation .

Ubiquitin-protein conjugation is increased in the diaphragmatic muscle of patients with mild to moderate COPD and this increase is associated with a loss of contractile protein and a decreased ability to generate force . The 20S proteasome activity is also increased in the diaphragm of patients with COPD and there is increased mRNA for a muscle-specific ubiquitin-ligase . COPD patients who have a low body mass index (<20 kg/m2) have also been shown to have an increase in apoptotic nuclei in their muscle

Despite the fact that the mechanisms may be different, our data raise the interesting speculation that smokers who develop emphysema-predominant COPD may have a genetic or acquired predisposition to excessive protein degradation and apoptosis in both their lung and peripheral tissues to explain the concordance of the two processes.

| **Case** | **FEV1 %P** | **COPD GOLD class** | **Core with Low S/V** | **Core with High S/V** | **Difference in S/V** |
| --- | --- | --- | --- | --- | --- |
|  |  |  | (cm2/mL) | (cm2/mL) | (cm2/mL) |
| 1 | 77 | 2 | 51 | 356 | 305 |
| 2 | 105 | 0 | 102 | 242 | 141 |
| 3 | 85 | 1 | 105 | 214 | 109 |
| 4 | 46 | 3 | 162 | 211 | 49 |
| 5 | 95 | 0 | 43 | 146 | 103 |
| 6 | 97 | 0 | 119 | 215 | 97 |
| 7 | 91 | 0 | 150 | 246 | 96 |
| 8 | 81 | 0 | 129 | 221 | 92 |
| 9 | 81 | 1 | 153 | 237 | 84 |
| 10 | 101 | 1 | 121 | 201 | 80 |
| 11 | 104 | 0 | 130 | 214 | 84 |
| 12 | 84 | 0 | 149 | 230 | 81 |
| 13 | 68 | 2 | 108 | 190 | 82 |
| 14 | 69 | 2 | 82 | 160 | 77 |
| 15 | 57 | 2 | 112 | 189 | 77 |
| 16 | 86 | 0 | 84 | 159 | 75 |
| 17 | 78 | 2 | 124 | 197 | 73 |
| 18 | 80 | 0 | 167 | 220 | 53 |
| 19 | 94 | 0 | 96 | 146 | 50 |
| 20 | 46 | 3 | 152 | 215 | 63 |
| 21 | 82 | 1 | 107 | 165 | 58 |

Table E1a. This table shows data for the 21 subjects or whom paired samples were used to generate a discover set of genes whose expression was related to lung surface to volume ratio (S/V cm^2^/mL). FEV1 as a percent predicted, GOLD class and the S/V ratios for the lung core with the low and high values are shown as well as the S/V ratio difference between the samples.

| **Case** | **FEV1%P** | **COPD GOLD class** | **S/V (cm2/mL)** |
| --- | --- | --- | --- |
|  |  |  |  |
| 1 | 63 | 2 | 323 |
| 2 | 94 | 0 | 301 |
| 3 | 94 | 1 | 255 |
| 4 | 123 | 0 | 230 |
| 5 | 90 | 1 | 201 |
| 6 | 61 | 2 | 199 |
| 7 | 93 | 0 | 191 |
| 8 | 99 | 3 | 186 |
| 9 | 118 | 0 | 177 |
| 10 | 96 | 0 | 172 |
| 11 | 92 | 0 | 171 |
| 12 | 86 | 0 | 169 |
| 13 | 96 | 0 | 154 |
| 14 | 93 | 0 | 152 |
| 15 | 109 | 0 | 148 |
| 16 | 86 | 1 | 146 |
| 17 | 65 | 2 | 135 |
| 18 | 123 | 0 | 129 |
| 19 | 46 | 3 | 117 |
| 20 | 97 | 1 | 104 |
| 21 | 86 | 1 | 97 |
| 22 | 61 | 2 | 93 |

Table E1b. This table shows data for the 22 subjects on whom only one lung sample was profiled serving as the replication sample for the relationship between gene expression and S/V ratio. The FEV1 as percent predicted, the COPD GOLD class and the S/V ratio of the profiled sample are shown.

|  | **CASE** | **Age** | **Diagnosis** | **Gender** | **FEV1/FVC ratio** | **FEV1 percent predicted** | **pkyrs** |
| --- | --- | --- | --- | --- | --- | --- | --- |
| **COPD** | 1 | 59 | BAC | F | 56 | 46 | 37 |
|  | 2 | 61 | COPD | F | 22 | 23 | NA |
|  | 3 | 57 | COPD | F | 23 | 19 | NA |
|  | 4 | 59 | COPD | F | 44 | 28 | 30 |
|  | 5 | 54 | COPD | F | 36. | 18 | 74 |
|  | 6 | 55 | COPD | M | 28 | 21 | 15 |
|  | 7 | 62 | COPD | M | 22 | 21 | 50 |
|  | 8 | 63 | COPD | F | 26 | 12 | 38 |
|  | 9 | 61 | COPD | F | 28 | 15 | 25 |
|  | 10 | 56 | COPD | F | 24 | 24 | 54 |
| **Control** | 1 | 43 | donor/CVA | M | NA | NA | 0 |
|  | 2 | 59 | donor/CVA | M | NA | NA | 0 |
|  | 3 | 62 | donor/arrest during CABG | M | NA | NA | 24 |
|  | 4 | 51 | donor/arrest | M | NA | NA | 39 |
|  | 5 | 36 | donor/head trauma | M | NA | NA | 20 |
|  | 6 | 69 | adenocarcinoma, irregular emphysema | M | 79 | 113 | 0 |
|  | 7 | 61 | carcinoid | F | 84 | 93 | 0 |
|  | 8 | 77 | adenocarcinoma | M | 79 | 110 | 92.5 |
|  | 9 | 74 | non-small cell | M | 70 | 88 | 130 |
|  | 10 | 47 | squamous cell carcinoma | F | 70 | 125 | 68 |
|  | 6348 | 65 | adenocarcinoma | F | 76 | 70 | 20 |

Table S3: Demographic and clinical details of subjects used for western blot analysis of ubiquitin pathway proteins. NA = not available. Pkyrs = pack years of smoking BAC =bronchoalveolar cell carcinoma, CVA =cardiovascular accident, CABG = coronary artery bypass grafting

|  | **Transcript** | **Genes** | **Validation slope** | **Validation p value** | **Training slope** |
| --- | --- | --- | --- | --- | --- |
| **1** | NM_005706 | TSSC4 | -0.000965 | 0.003232 | -0.000369 |
| **2** | AB067498 | EFO1,KIAA1911 | 0.000499 | 0.004945 | 0.000381 |
| **3** | NM_018559 | KIAA1704,LSR7,AD029,bA245H20.2 | 0.000648 | 0.005959 | 0.000404 |
| **4** | NM_004897 | MINPP1,MIPP,HIPER1,MINPP2,DKFZp564L2016 | 0.000698 | 0.006042 | 0.000353 |
| **5** | Contig55580_RC |  | 0.001390 | 0.006252 | 0.000487 |
| **6** | NM_012180 | FBXO8,FBS,DC10,FBX8 | 0.000665 | 0.013920 | 0.000398 |
| **7** | NM_153044 | FLJ35801 | -0.000543 | 0.015270 | -0.000402 |
| **8** | NM_022876 | SMN2,SMNC,BCD541,C-BCD541 | 0.000828 | 0.016530 | 0.000456 |
| **9** | NM_003838 | FPGT,GFPP | 0.000538 | 0.018290 | 0.000353 |
| **10** | AF055030 | PHF10,XAP135,FLJ10975 | 0.000666 | 0.018770 | 0.000362 |
| **11** | NM_007342 | NUPL2,CG1,hCG1,NLP-1,NLP_1 | 0.000805 | 0.019200 | 0.000365 |
| **12** | Contig45624_RC | CBLL1 | 0.000912 | 0.019740 | 0.000517 |
| **13** | NM_002103 | GYS1,GSY,GYS | -0.000519 | 0.019950 | -0.000333 |
| **14** | AL359938 | MEIS3,MRG2,DKFZp547H236 | -0.000535 | 0.022060 | -0.000294 |
| **15** | NM_014771 | RNF40,BRE1B,RBP95,STARING,KIAA0661,MGC13051 | -0.000668 | 0.024170 | -0.000337 |
| **16** | NM_022877 | SMN2,SMNC,BCD541,C-BCD541 | 0.000772 | 0.024290 | 0.000465 |
| **17** | Contig41498_RC | PTPN4,PTPMEG,PTPMEG1 | 0.000492 | 0.024360 | 0.000348 |
| **18** | NM_003084 | SNAPC3,SNAP50,PTFbeta | 0.000747 | 0.025220 | 0.000445 |
| **19** | NM_013234 | eIF3k,M9,ARG134,PTD001,HSPC029,MSTP001,PLAC-24,PRO1474 | -0.000452 | 0.026540 | -0.000254 |
| **20** | NM_006852 | TLK2,MGC44450,PKU-ALPHA | 0.000322 | 0.027710 | 0.000209 |
| **21** | X68560 | SP3,SPR-2 | 0.001132 | 0.029570 | 0.000647 |
| **22** | AY007149 | CAP350,gm133,KIAA0480 | 0.000582 | 0.030350 | 0.000345 |
| **23** | Contig51940_RC | GABPA,NFT2,NRF2,NRF2A,E4TF1A,E4TF1-60 | 0.000931 | 0.032800 | 0.000458 |
| **24** | NM_032557 | USP38,HP43.8KD,KIAA1891 | 0.000731 | 0.034130 | 0.000477 |
| **25** | NM_015153 | PHF3,KIAA0244 | 0.000574 | 0.036450 | 0.000599 |
| **26** | NM_004162 | RAB5A,RAB5 | 0.000477 | 0.036500 | 0.000300 |
| **27** | NM_022875 | SMN2,SMNC,BCD541,C-BCD541 | 0.000709 | 0.038730 | 0.000484 |
| **28** | NM_005316 | GTF2H1,BTF2,TFIIH | 0.000690 | 0.038870 | 0.000423 |
| **29** | Contig53191_RC | GPD2,GDH2 | 0.000686 | 0.041270 | 0.000432 |
| **30** | NM_017411 | SMN2,SMNC,BCD541,C-BCD541 | 0.000707 | 0.043600 | 0.000477 |
| **31** | NM_006950 | SYN1,SYNI,SYN1a,SYN1b | -0.000724 | 0.055360 | -0.000399 |
| **32** | NM_138290 | RPIB9,RPIP9,FLJ30671,MGC26655 | -0.001156 | 0.062960 | -0.000549 |
| **33** | Contig51328_RC | DKFZp434H2226 | 0.000879 | 0.064140 | 0.000534 |
| **34** | Contig49468_RC | RSC1A1,RS1 | 0.000904 | 0.065120 | 0.000443 |
| **35** | Contig43027_RC | ING3 | 0.000573 | 0.065880 | 0.000467 |
| **36** | NM_014812 | KAB,KIAA0470 | 0.000376 | 0.066020 | 0.000364 |
| **37** | X15624 | RPPH1,H1RNA | -0.001133 | 0.066540 | -0.000887 |
| **38** | NM_016374 | ARID4B,BCAA,BRCAA1,RBP1L1,SAP180,RBBP1L1 | 0.000682 | 0.066820 | 0.000417 |
| **39** | AL049378 | TNPO1,MIP,TRN,IPO2,MIP1,KPNB2 | 0.000787 | 0.068730 | 0.000449 |
| **40** | AF054589 | HIC | 0.000969 | 0.070560 | 0.000558 |
| **41** | NM_002890 | RASA1,GAP,PKWS,RASA,CMAVM,RASGAP,p120GAP | 0.000716 | 0.071590 | 0.000460 |
| **42** | NM_018448 | TIP120A,CAND1,TIP120,KIAA0829 | 0.000677 | 0.071880 | 0.000348 |
| **43** | NM_005977 | RNF6 | 0.000659 | 0.073010 | 0.000364 |
| **44** | Contig8909_RC | TAB3 | 0.000328 | 0.086390 | 0.000380 |
| **45** | Contig54110_RC | SFRS1,ASF,SF2,SRp30a | 0.000761 | 0.089520 | 0.000629 |
| **46** | AL049951 | NEXN,NELIN,nexilin | 0.000638 | 0.090100 | 0.000556 |
| **47** | NM_018330 | KIAA1598 | 0.000676 | 0.090690 | 0.000540 |
| **48** | NM_013450 | BAZ2B,DKFZP434H071 | 0.000434 | 0.093140 | 0.000367 |
| **49** | AL137753 | KIAA1033 | 0.000676 | 0.095210 | 0.000627 |
| **50** | NM_019593 | KIAA1434,FLJ11085,MGC26147 | 0.000935 | 0.101100 | 0.000589 |
| **51** | NM_012117 | CBX5,HP1,HP1-ALPHA,HP1Hs-alpha | -0.001111 | 0.102000 | -0.000685 |
| **52** | AL133033 | THRAP2,MED13L,KIAA1025,TRAP240L,PROSIT240 | 0.000682 | 0.102400 | 0.000359 |
| **53** | Contig46646_RC |  | 0.000786 | 0.103600 | 0.000475 |
| **54** | AK055661 | ZBTB34,KIAA1993,MGC24652 | 0.000448 | 0.104000 | 0.000569 |
| **55** | NM_013386 | SLC25A24,APC1,SCAMC-1,DKFZp586G0123 | 0.000501 | 0.109100 | 0.000601 |
| **56** | AJ457796 | SPANXF1 | -0.001017 | 0.112600 | -0.000755 |
| **57** | NM_001533 | HNRPL,hnRNP-L,P/OKcl.14 | -0.000410 | 0.112700 | -0.000267 |
| **58** | Contig45995_RC | FLJ35258 | -0.000684 | 0.113600 | -0.000607 |
| **59** | NM_004270 | CRSP9,MED7,CRSP33,MGC12284 | 0.000256 | 0.115600 | 0.000239 |
| **60** | Contig45544_RC | PRKRA | 0.000793 | 0.115800 | 0.000689 |
| **61** | Contig37371_RC |  | 0.000335 | 0.120800 | 0.000456 |
| **62** | NM_015358 | ZCWCC3,NXP2,ZCW5,KIAA0136 | 0.000728 | 0.120800 | 0.000512 |
| **63** | Contig39177_RC | LOC284267 | 0.000411 | 0.122400 | 0.000375 |
| **64** | NM_030921 | TBL1XR1,C21,DC42,IRA1,TBLR1,FLJ12894 | 0.001129 | 0.126000 | 0.000688 |
| **65** | Contig50355_RC | AVO3,Rictor,KIAA1999,MGC39830 | 0.000579 | 0.126700 | 0.000448 |
| **66** | NM_032124 | HDHD2,3110052N05Rik,DKFZP564D1378 | 0.000297 | 0.135400 | 0.000348 |
| **67** | NM_014999 | RAB21,KIAA0118 | 0.000714 | 0.146400 | 0.000431 |
| **68** | NM_052857 | MGC20398 | 0.000465 | 0.148200 | 0.000392 |
| **69** | NM_002805 | PSMC5,S8,p45,SUG1,TBP10,TRIP1,p45/SUG | -0.000267 | 0.150800 | -0.000262 |
| **70** | NM_017827 | SARS2,SYS,SARS,SERS,SARSM,SerRSmt,mtSerRS,FLJ20450 | -0.000488 | 0.152800 | -0.000316 |
| **71** | NM_019083 | FLJ10287,FLJ11219 | 0.000650 | 0.156100 | 0.000486 |
| **72** | NM_031310 | PLVAP,PV1,FELS,PV-1,gp68 | -0.000883 | 0.156100 | -0.001101 |
| **73** | Contig49578_RC | FLJ20274 | 0.000324 | 0.157300 | 0.000309 |
| **74** | NM_005809 | PRDX2,PRP,TSA,NKEFB,PRXII,TDPX1,MGC4104 | -0.000349 | 0.157400 | -0.000235 |
| **75** | AL080216 | TRIM33,PTC7,RFG7,TF1G,TIF1G,FLJ11429,KIAA1113,TIFGAMMA,TIF1GAMMA | 0.000708 | 0.162400 | 0.000659 |
| **76** | NM_178225 | FBXW5,Fbw5,MGC20962,DKFZP434B205 | -0.000430 | 0.164900 | -0.000475 |
| **77** | NM_015938 | CGI-07,NMD3 | 0.000321 | 0.165100 | 0.000334 |
| **78** | NM_177948 | ARMCX3,ALEX3,MGC12199,dJ545K15.2 | -0.000401 | 0.166800 | 0.000335 |
| **79** | NM_033087 | ALG2,CDGIi,hALPG2,FLJ14511 | 0.000492 | 0.169700 | 0.000326 |
| **80** | NM_145342 | MAP3K7IP2,TAB2,FLJ21885,KIAA0733 | 0.000377 | 0.174900 | 0.000300 |
| **81** | NM_031296 | RAB33B,DKFZP434G099 | 0.000505 | 0.183100 | 0.000515 |
| **82** | NM_033224 | PURB,PURBETA | -0.000560 | 0.187000 | -0.000432 |
| **83** | NM_005359 | SMAD4,JIP,DPC4,MADH4 | 0.000518 | 0.188000 | 0.000438 |
| **84** | NM_002475 | MLC1SA | -0.000388 | 0.192900 | -0.000384 |
| **85** | NM_052969 | RPL39L,RPL39L1 | 0.000576 | 0.196100 | -0.000575 |
| **86** | AB033066 | KIAA1240 | 0.000390 | 0.199600 | 0.000490 |
| **87** | NM_016248 | AKAP11,PRKA11,AKAP220,FLJ11304,KIAA0629 | 0.000410 | 0.209800 | 0.000466 |
| **88** | AL050205 | LOC113251,PP13296 | 0.000532 | 0.215900 | 0.000481 |
| **89** | NM_020317 | NPD014,DJ465N24.2.1 | 0.000409 | 0.221300 | 0.000355 |
| **90** | NM_015344 | LEPROTL1,my047 | 0.000284 | 0.225800 | 0.000487 |
| **91** | NM_005186 | CAPN1,CANP,muCL,CANPL1,muCANP | -0.000435 | 0.240100 | -0.000380 |
| **92** | NM_019591 | ZNF26,KOX20,FLJ20755 | 0.000351 | 0.241100 | 0.000442 |
| **93** | NM_153331 | KCTD6,MGC27385 | 0.000318 | 0.244200 | 0.000285 |
| **94** | NM_030621 | DICER1,Dicer,HERNA,KIAA0928 | 0.000443 | 0.246100 | 0.000403 |
| **95** | Contig23423_RC |  | 0.000319 | 0.248500 | 0.000435 |
| **96** | NM_012158 | FBXL3,FBL3,FBL3A,FBXL3A | 0.000471 | 0.249600 | 0.000442 |
| **97** | NM_015925 | LISCH7,LSR,MGC10659,MGC48312 | 0.000473 | 0.250200 | -0.000284 |
| **98** | NM_015937 | PIGT,CGI-06,MGC8909 | 0.000332 | 0.254600 | -0.000357 |
| **99** | AL110180 |  | 0.000708 | 0.257300 | 0.000665 |
| **100** | NM_014739 | BCLAF1,BTF,KIAA0164,bK211L9.1 | 0.000228 | 0.260300 | 0.000309 |
| **101** | NM_177947 | ARMCX3,ALEX3,MGC12199,dJ545K15.2 | -0.000301 | 0.267200 | 0.000317 |
| **102** | NM_018368 | C6orf209,FLJ11240,bA810I22.1 | 0.000460 | 0.272000 | 0.000439 |
| **103** | NM_152270 | FLJ34922 | -0.000352 | 0.300500 | 0.000457 |
| **104** | NM_018955 | UBB,MGC8385 | 0.000469 | 0.300700 | 0.000434 |
| **105** | NM_020654 | SENP7,KIAA1707 | 0.000426 | 0.301400 | 0.000461 |
| **106** | NM_152995 | HOZFP | 0.000332 | 0.302000 | 0.000410 |
| **107** | NM_004719 | SFRS2IP,SIP1,CASP11,SRRP129 | 0.000237 | 0.304200 | 0.000422 |
| **108** | NM_003297 | NR2C1,TR2,TR2-11 | 0.000360 | 0.304300 | 0.000367 |
| **109** | Contig54274_RC | HSPC063 | 0.000355 | 0.307600 | 0.000459 |
| **110** | Contig52881_RC | KIAA1982 | 0.000391 | 0.318100 | 0.000471 |
| **111** | NM_014936 | ENPP4,NPP4,KIAA0879 | 0.000393 | 0.319800 | 0.000723 |
| **112** | Contig25653_RC | CDH11,OB,CAD11,CDHOB,OSF-4 | 0.000730 | 0.323300 | 0.000721 |
| **113** | Contig41169_RC | SLC25A30 | 0.000390 | 0.333700 | 0.000527 |
| **114** | NM_032145 | FBXO30,Fbx30,MGC21674 | 0.000289 | 0.350700 | 0.000417 |
| **115** | NM_004064 | CDKN1B,KIP1,CDKN4,P27KIP1 | 0.000368 | 0.355100 | 0.000545 |
| **116** | NM_003100 | SNX2,MGC5204 | 0.000356 | 0.362300 | 0.000425 |
| **117** | NM_022771 | TBC1D15,FLJ12085,DKFZp761D0223 | 0.000341 | 0.365100 | 0.000466 |
| **118** | NM_014845 | KIAA0274,SAC3,hSac3,dJ249I4.1 | -0.000363 | 0.374900 | 0.000410 |
| **119** | NM_080748 | C20orf52,bA353C18.2 | -0.000263 | 0.382200 | -0.000251 |
| **120** | NM_024937 | LRP12,ST7,FLJ12929 | 0.000384 | 0.384600 | 0.000414 |
| **121** | NM_016090 | RBM7 | 0.000337 | 0.389700 | 0.000579 |
| **122** | ENST00000293904 | ENST00000293904 | -0.000229 | 0.389800 | -0.000368 |
| **123** | NM_153207 | AEBP2,MGC17922 | 0.000330 | 0.395300 | 0.000381 |
| **124** | NM_020771 | HACE1 | 0.000383 | 0.410200 | 0.000516 |
| **125** | NM_015323 | KIAA0776 | 0.000269 | 0.418800 | 0.000342 |
| **126** | Contig46615_RC | LOC124491 | 0.000195 | 0.426100 | 0.000352 |
| **127** | Contig57877_RC | PRKAA1,MGC33776,MGC57364 | 0.000194 | 0.426800 | 0.000378 |
| **128** | Contig53804_RC | ARRDC4 | -0.000420 | 0.432700 | 0.000704 |
| **129** | NM_003663 | CGGBP1,CGGBP,p20-CGGBP | 0.000253 | 0.439500 | 0.000316 |
| **130** | NM_015571 | SENP6,SSP1,SUSP1,KIAA0797 | 0.000310 | 0.443600 | 0.000434 |
| **131** | NM_006055 | LANCL1,p40,GPR69A | 0.000209 | 0.445600 | 0.000353 |
| **132** | NM_153453 | VGLL2,VGL2,VITO1 | -0.000249 | 0.448100 | -0.000383 |
| **133** | NM_000153 | GALC | 0.000177 | 0.460200 | 0.000591 |
| **134** | NM_152434 | CWF19L2,FLJ32343 | 0.000160 | 0.461600 | 0.000264 |
| **135** | NM_000858 | GUK1,GMK | -0.000201 | 0.462800 | -0.000346 |
| **136** | NM_000950 | PRRG1,PRGP1 | 0.000484 | 0.463300 | 0.000812 |
| **137** | NM_020666 | CLK4 | 0.000293 | 0.469100 | 0.000493 |
| **138** | NM_144658 | DOCK11,FLJ32122,FLJ43653,bB128O4.1 | 0.000255 | 0.479400 | 0.000506 |
| **139** | NM_003972 | BTAF1,MOT1,TAF172,TAFII170,TAF(II)170 | 0.000312 | 0.482000 | 0.000516 |
| **140** | NM_024811 | FLJ12529,MGC9315,FLJ39024 | -0.000202 | 0.482400 | 0.000367 |
| **141** | AK056521 | BTBD14A,BTBD14,MGC23427 | -0.000283 | 0.492700 | -0.000550 |
| **142** | ENST00000284414 | ENST00000284414 | -0.000229 | 0.504400 | -0.000388 |
| **143** | Contig2745_RC | SELT | 0.000189 | 0.517600 | 0.000387 |
| **144** | ENST00000285726 | ENST00000285726 | -0.000392 | 0.532000 | -0.000732 |
| **145** | AL050064 | RSBN1,ROSBIN | 0.000177 | 0.540700 | 0.000434 |
| **146** | NM_017822 | FLJ20436,FLJ12670 | 0.000129 | 0.563400 | 0.000312 |
| **147** | NM_017742 | ZCCHC2,FLJ20281,KIAA1744,MGC13269,DKFZp451A185 | 0.000321 | 0.567000 | 0.000805 |
| **148** | Contig50388_RC | ZNF148,BERF-1,BFCOL1,ZBP-89,ZFP148,pHZ-52,HT-BETA | 0.000177 | 0.567400 | 0.000375 |
| **149** | Contig749_RC | HSPC063 | 0.000222 | 0.568100 | 0.000501 |
| **150** | NM_152306 | UHRF2,NIRF,URF2,RNF107,MGC33463 | 0.000217 | 0.573400 | 0.000428 |
| **151** | Contig1277 | COL4A3BP,CERT,GPBP,CERTL,STARD11 | 0.000123 | 0.580000 | 0.000312 |
| **152** | NM_015423 | AASDHPPT,LYS2,LYS5,CGI-80,AASD-PPT,DKFZp566E2346 | 0.000194 | 0.585600 | 0.000294 |
| **153** | Contig50396_RC | LOC339924 | 0.000247 | 0.589300 | 0.000613 |
| **154** | NM_016141 | DNCLI1 | 0.000114 | 0.628200 | 0.000361 |
| **155** | Contig48185_RC | DDHD1 | 0.000176 | 0.634700 | 0.000537 |
| **156** | Contig35435_RC | FLJ23447 | -0.000104 | 0.636400 | -0.000238 |
| **157** | ENST00000296831 | ENST00000296831 | -0.000064 | 0.640500 | -0.000163 |
| **158** | NM_014912 | CPEB3 | 0.000190 | 0.641800 | 0.000531 |
| **159** | NM_004788 | UBE4A,E4,UFD2,KIAA0126 | 0.000090 | 0.651200 | 0.000197 |
| **160** | NM_015288 | PHF15,JADE2,KIAA0239 | -0.000086 | 0.666200 | -0.000364 |
| **161** | NM_153261 | FLJ38101 | 0.000137 | 0.704100 | 0.000331 |
| **162** | NM_133462 | TTC14,FLJ00166,KIAA1980 | 0.000136 | 0.706100 | 0.000474 |
| **163** | NM_017900 | AKIP,AIP,FLJ20608 | -0.000123 | 0.710500 | -0.000282 |
| **164** | Contig53364_RC | SCN7A,SCN6A | -0.000267 | 0.713000 | 0.000917 |
| **165** | Contig31559_RC |  | 0.000150 | 0.723200 | 0.000553 |
| **166** | ENST00000300012 | ENST00000300012 | 0.000184 | 0.728800 | -0.000421 |
| **167** | NM_005037 | PPARG,NR1C3,PPARG1,PPARG2,HUMPPARG | -0.000233 | 0.773900 | 0.000890 |
| **168** | NM_018133 | FLJ10546,KIAA1585 | 0.000089 | 0.808900 | 0.000479 |
| **169** | NM_004593 | SFRS10,TRA2B,SRFS10,TRA2-BETA,Htra2-beta | 0.000058 | 0.837600 | 0.000330 |
| **170** | NM_018061 | FLJ10330 | 0.000054 | 0.852700 | 0.000349 |
| **171** | NM_030773 | TUBB1,dJ543J19.4 | -0.000044 | 0.855500 | -0.000465 |
| **172** | NM_016607 | ARMCX3,ALEX3,MGC12199,dJ545K15.2 | -0.000069 | 0.856900 | 0.000473 |
| **173** | Contig31615_RC | LOC339692 | -0.000046 | 0.861500 | -0.000393 |
| **174** | NM_177949 | ARMCX2,ALEX2,MGC8742,KIAA0512,MGC13343 | -0.000065 | 0.897900 | 0.000447 |
| **175** | Contig44180_RC |  | -0.000068 | 0.903700 | -0.000705 |
| **176** | NM_152634 | MGC17403 | 0.000031 | 0.904000 | 0.000252 |
| **177** | NM_007080 | LSM6,YDR378C | -0.000023 | 0.909400 | 0.000439 |
| **178** | NM_172193 | KLHDC1,MST025,c14_5298 | 0.000045 | 0.911100 | 0.000427 |
| **179** | Contig41828_RC |  | -0.000022 | 0.925300 | 0.000321 |
| **180** | NM_002975 | SCGF,P47,LSLCL,CLECSF3 | -0.000045 | 0.926700 | -0.000633 |
| **181** | NM_015869 | PPARG,NR1C3,PPARG1,PPARG2,HUMPPARG | -0.000017 | 0.982500 | 0.001043 |

Table S4: 181 SA/V – related genes

| # | **Processes** | **p-value** |
| --- | --- | --- |
| 1 | cellular macromolecule metabolic process | 1.417E-10 |
| 2 | macromolecule metabolic process | 2.857E-09 |
| 3 | metabolic process | 5.535E-09 |
| 4 | regulation of RNA metabolic process | 1.706E-08 |
| 5 | ubiquitin-dependent protein catabolic process | 2.103E-08 |
| 6 | regulation of cellular macromolecule biosynthetic process | 2.356E-08 |
| 7 | modification-dependent protein catabolic process | 2.635E-08 |
| 8 | protein modification by small protein conjugation | 2.667E-08 |
| 9 | modification-dependent macromolecule catabolic process | 3.054E-08 |
| 10 | primary metabolic process | 4.023E-08 |

Table S5. The top 10 gene ontology (GO) processes enriched in the 181 genes.

| **Transcript ID** | **Symbol** | **Description** | **Cellular function** |
| --- | --- | --- | --- |
| NM_012158 | FBXL3 | F-box and leucine-rich repeat protein 3 | A component of the ubiquitin ligase complex SCFs (SKP1-cullin-F-box) |
| NM_032145 | FBXO30 | F-box protein 30 | A component of the ubiquitin ligase complex SCFs (SKP1-cullin-F-box) |
| NM_032557 | USP38 | Ubiquitin specific protease 38 | Deubiquitinating enzyme |
| NM_108955 | UBB | Ubiquitin B | Polyubiquitin precursor protein |
| NM_005977 | RNF6 | RING (Really Interesting New Gene) finger protein 6 | RING finger domain ubiquitin ligase |
| NM_018133 | RNF184 | RING finger protein 184 | RING finger domain ubiquitin ligase |
| NM_004788 | UBE4A | Ubiquitination factor E4A | U-box-type ubiquitin ligase |
| NM_152306 | UHRF2 | Ubiquitin-like, containing PHD and RING finger domain, 2 | Ubiquitin ligase |
| NM_030921 | TBLR1 | Transducin β-Like Receptor 1 | Adaptor protein for the recruitment of the ubiquitin conjugating/19S proteasome complex |

Table S6. The 9 ubiquitination-associated genes that were interrogated using Western Blotting


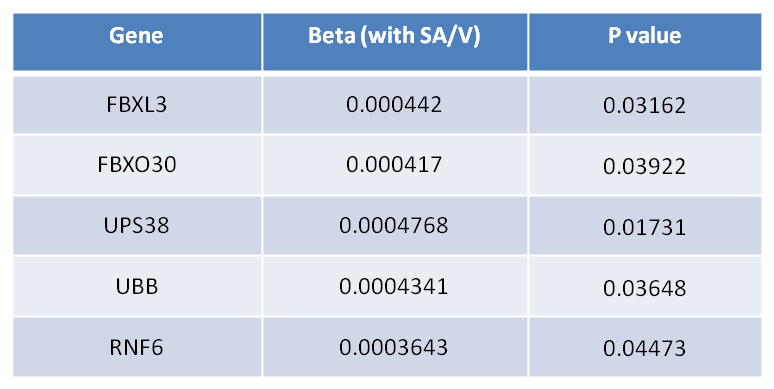


Table S7. The relationship of the level of expression of ubiquitination-associated genes and SA/V in the training set.

Figure S1: One-dimensional clustering using bright and variable genes indicates a significant batch effect between Phase 1 and 2 data. The number of genes significantly different between the two data sets using ANOVA p < 0.01 equals ~14000.

Figure S2: Corresponding sample pairs co-cluster after preprocessing. This indicates that the preprocessing is valid and shows that there are many patient specific genes, i.e. genes that vary more between than within individuals.

**References:**

1. Wang IM, Stepaniants S, Boie Y, Mortimer JR, Kennedy B, Elliott M, Hayashi S, Loy L, Coulter S, Cervino S, Harris J, Thornton M, Raubertas R, Roberts C, Hogg JC, Crackower M, O'Neill G, Paré PD. Gene Expression Profiling in Patients with Chronic Obstructive Pulmonary Disease and Lung Cancer. *American Journal of Respiratory and Critical Care Medicine* 2008: 177(4): 402-411.

2. Hankinson JL, Odencrantz JR, Fedan KB. Spirometric reference values from a sample of the general U.S. population. *Am J Respir Crit Care Med* 1999: 159(1): 179-187.

3. Crapo RO, Morris AH. Standardized single breath normal values for carbon monoxide diffusing capacity. *Am Rev Respir Dis* 1981: 123(2): 185-189.

4. Luo H, Zhang J, Cheung C, Suarez A, McManus BM, Yang D. Proteasome Inhibition Reduces Coxsackievirus B3 Replication in Murine Cardiomyocytes. *The American journal of pathology* 2003: 163(2): 381-385.

5. Rauvala H, Rouhiainen A. Physiological and pathophysiological outcomes of the interactions of HMGB1 with cell surface receptors. *Biochimica et Biophysica Acta (BBA) - Gene Regulatory Mechanisms* 2010: 1799(1–2): 164-170.

6. Repapi E, Sayers I, Wain LV, Burton PR, Johnson T, Obeidat Me, Zhao JH, Ramasamy A, Zhai G, Vitart V, Huffman JE, Igl W, Albrecht E, Deloukas P, Henderson J, Granell R, McArdle WL, Rudnicka AR, Barroso I, Loos RJF, Wareham NJ, Mustelin L, Rantanen T, Surakka I, Imboden M, Wichmann HE, Grkovic I, Jankovic S, Zgaga L, Hartikainen A-L, Peltonen L, Gyllensten U, Johansson A, Zaboli G, Campbell H, Wild SH, Wilson JF, Glaser S, Homuth G, Volzke H, Mangino M, Soranzo N, Spector TD, Polasek O, Rudan I, Wright AF, Heliovaara M, Ripatti S, Pouta A, Naluai AT, Olin A-C, Toren K, Cooper MN, James AL, Palmer LJ, Hingorani AD, Wannamethee SG, Whincup PH, Smith GD, Ebrahim S, McKeever TM, Pavord ID, MacLeod AK, Morris AD, Porteous DJ, Cooper C, Dennison E, Shaheen S, Karrasch S, Schnabel E, Schulz H, Grallert H, Bouatia-Naji N, Delplanque J, Froguel P, Blakey JD, Britton JR, Morris RW, Holloway JW, Lawlor DA, Hui J, Nyberg F, Jarvelin M-R, Jackson C, Kahonen M, Kaprio J, Probst-Hensch NM, Koch B, Hayward C, Evans DM, Elliott P, Strachan DP, Hall IP, Tobin MD. Genome-wide association study identifies five loci associated with lung function. *Nat Genet* 2010: 42(1): 36-44.

7. Yu M, Wang H, Ding A, Golenbock DT, Latz E, Czura CJ, Fenton MJ, Tracey KJ, Yang H. Hmgb1 Signals Through Toll-Like Receptor (Tlr) 4 and Tlr2. *Shock* 2006: 26(2): 174-179 110.1097/1001.shk.0000225404.0000251320.0000225482.

8. Lafferty E, Qureshi S, Schnare M. The role of toll-like receptors in acute and chronic lung inflammation. *Journal of Inflammation* 2010: 7(1): 57.

9. Bezemer GFG, Sagar S, van Bergenhenegouwen J, Georgiou NA, Garssen J, Kraneveld AD, Folkerts G. Dual Role of Toll-Like Receptors in Asthma and Chronic Obstructive Pulmonary Disease. *Pharmacological Reviews* 2012: 64(2): 337-358.

10. Coxson HO, Chan IHT, Mayo JR, Hlynsky J, Nakano Y, Birmingham CL. Early Emphysema in Patients with Anorexia Nervosa. *American Journal of Respiratory and Critical Care Medicine* 2004: 170(7): 748-752.

11. Haehling S, Anker S. Cachexia as a major underestimated and unmet medical need: facts and numbers. *J Cachexia Sarcopenia Muscle* 2010: 1(1): 1-5.

12. Ogawa E, Nakano Y, Ohara T, Muro S, Hirai T, Sato S, Sakai H, Tsukino M, Kinose D, Nishioka M, Niimi A, Chin K, Paré PD, Mishima M. Body mass index in male patients with COPD: correlation with low attenuation areas on CT. *Thorax* 2009: 64(1): 20-25.

13. Kurosaki H, Ishii T, Motohashi N, Motegi T, Yamada K, Kudoh S, Jones RCM, Kida K. Extent of Emphysema on HRCT Affects Loss of Fat-free Mass and Fat Mass in COPD. *Internal Medicine* 2009: 48(1): 41-48.

14. Hansen MJ, Gualano RC, Bozinovski S, Vlahos R, Anderson GP. Therapeutic prospects to treat skeletal muscle wasting in COPD (chronic obstructive lung disease). *Pharmacology & Therapeutics* 2006: 109(1–2): 162-172.

15. Ottenheijm CA, Heunks LM, Dekhuijzen RP. Diaphragm adaptations in patients with COPD. *Respir Res* 2008: 9: 12.

16. Remels AH, Gosker HR, Langen RC, Schols AM. The mechanisms of cachexia underlying muscle dysfunction in COPD. *J Appl Physiol* 2012.

17. Wust RC, Degens H. Factors contributing to muscle wasting and dysfunction in COPD patients. *Int J Chron Obstruct Pulmon Dis* 2007: 2(3): 289-300.

18. Ottenheijm CA, Heunks LM, Li YP, Jin B, Minnaard R, van Hees HW, Dekhuijzen PN. Activation of the ubiquitin-proteasome pathway in the diaphragm in chronic obstructive pulmonary disease. *Am J Respir Crit Care Med* 2006: 174(9): 997-1002.

19. Ottenheijm CA, Heunks LM, Sieck GC, Zhan WZ, Jansen SM, Degens H, de Boo T, Dekhuijzen PN. Diaphragm dysfunction in chronic obstructive pulmonary disease. *Am J Respir Crit Care Med* 2005: 172(2): 200-205.

20. Debigare R, Cote CH, Maltais F. Ubiquitination and proteolysis in limb and respiratory muscles of patients with chronic obstructive pulmonary disease. *Proc Am Thorac Soc* 2010: 7(1): 84-90.

21. Eddins MJ, Marblestone JG, Suresh Kumar KG, Leach CA, Sterner DE, Mattern MR, Nicholson B. Targeting the ubiquitin E3 ligase MuRF1 to inhibit muscle atrophy. *Cell Biochem Biophys* 2011: 60(1-2): 113-118.

22. Ottenheijm CAC, Heunks LMA, Sieck GC, Zhan W-Z, Jansen SM, Degens H, de Boo T, Dekhuijzen PNR. Diaphragm Dysfunction in Chronic Obstructive Pulmonary Disease. *American Journal of Respiratory and Critical Care Medicine* 2005: 172(2): 200-205.

23. Ottenheijm CAC, Heunks LMA, Li Y-P, Jin B, Minnaard R, van Hees HWH, Dekhuijzen PNR. Activation of the Ubiquitin–Proteasome Pathway in the Diaphragm in Chronic Obstructive Pulmonary Disease. *American Journal of Respiratory and Critical Care Medicine* 2006: 174(9): 997-1002.

24. Agustí AGN, Sauleda J, Miralles C, Gomez C, Togores B, Sala E, Batle S, Busquets X. Skeletal Muscle Apoptosis and Weight Loss in Chronic Obstructive Pulmonary Disease. *American Journal of Respiratory and Critical Care Medicine* 2002: 166(4): 485-489.
