# Supplementary figures and images for: Genes related to emphysema are enriched for ubiquitination pathways
Source: BMC Pulm Med. 2014 Nov 29;14:187. doi: 10.1186/1471-2466-14-187 (PMC4280711; doi:10.1186/1471-2466-14-187)

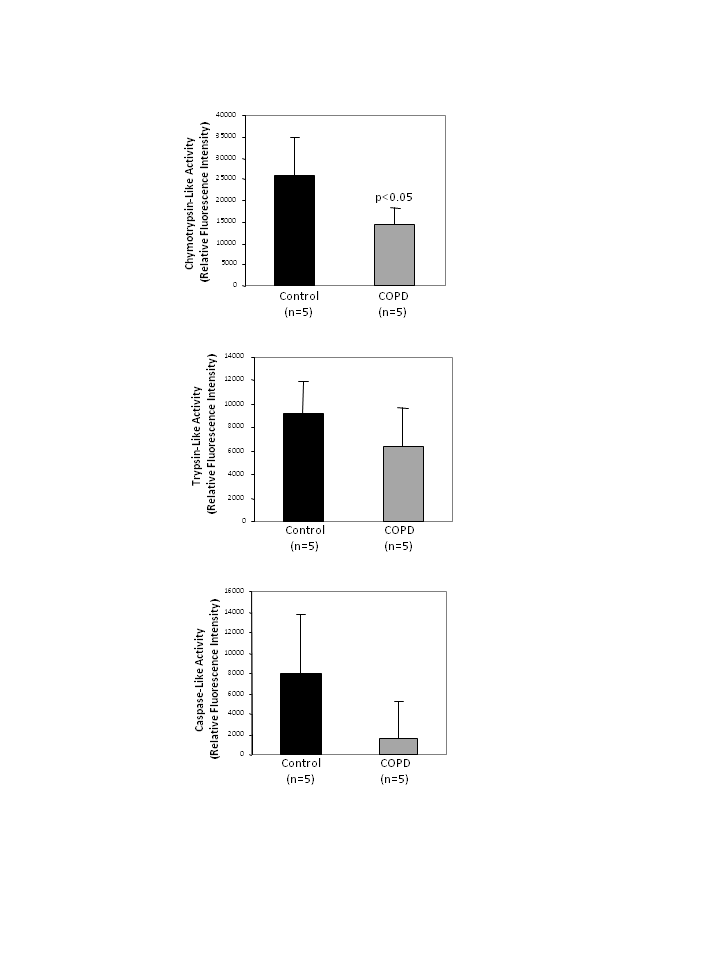

Supplement: Supplementary file 2 — Additional file 2: Figure S3: Proteasome activities in lung tissues from control and COPD patients. Lung homogenates were prepared and chymotrypsin-, trypsin-, and caspase-like proteasome activities were measured as described in the Materials and Methods. Results are expressed as means ± SE. Significance was determined by Student's t-test. (TIFF 71 KB) [file 12890_2014_636_MOESM2_ESM.tiff]
